# Genomic analysis of *Elsinoë arachidis* reveals its potential pathogenic mechanism and the biosynthesis pathway of elsinochrome toxin

**DOI:** 10.1371/journal.pone.0261487

**Published:** 2021-12-16

**Authors:** Wenli Jiao, Mengxue Xu, Rujun Zhou, Yiwei Fu, Zibo Li, Caiyun Xue

**Affiliations:** Department of Plant Pathology, College of Plant Protection, Shenyang Agricultural University, Shenyang, Liaoning, China; Fujian Agriculture and Forestry University, CHINA

## Abstract

Elsinochromes (ESCs) are virulence factors produced by *Elsinoë arachidis* which is the cause of peanut scab. However, the biosynthesis pathway of ESCs in *E*. *arachidis* has not been elucidated and the potential pathogenic mechanism of *E*. *arachidis* is poorly understood. In this study, we report a high-quality genome sequence of *E*. *arachidis*. The size of the *E*. *arachidis* genome is 33.18Mb, which is comparable to the Ascomycota genome (average 36.91 Mb), encoding 9174 predicted genes. The self-detoxification family including transporters and cytochrome P450 enzymes were analysis, candidate effectors and cell wall degrading enzymes were investigated as the pathogenicity genes by using PHI and CAZy databases. Additionally, the *E*. *arachidis* genome contains 24 secondary metabolism gene clusters, in which *ESCB1* was identified as the core gene of ESC biosynthesis. Taken together, the genome sequence of *E*. *arachidis* provides a new route to explore its potential pathogenic mechanism and the biosynthesis pathway of ESCs.

## Introduction

*Elsinoë arachidis* is a phytopathogenic fungus that causes peanut scab on *Arachis hypogaea* Linn., resulting in tremendous yield loss (regional losses can be greater than 50%) in peanut planting regions in China [1, 2]. Currently, disease occurrence patterns have been determined. However, the mechanism of host-pathogen interactions is largely unknown, indicating that new and effective prevention and control mechanisms of *E*. *arachidis* are urgently needed [3–6].

Interestingly, several *Elsinoë* produce elsinochromes (ESCs) [7], which are red, photosensitive, perylenequinone toxins. Previously, ESCs have been shown to promote electrolyte leakage, peroxidation of the plasma membrane, and production of reactive oxygen species such as superoxide (O_2_^–^). Additionally, ESCs contribute to pathogenesis and are essential for full virulence which was validated by constructing mutants in *E*. *fawcettii* of a polyketide synthase-encoding gene which is the core gene of ESC biosynthesis [8–10]. Cercosporin (*Cercospora* spp.) is the most well-known member of the group of perylenequinone fungal toxins. The biological functions and biosynthetic pathway of cercosporin have been clarified. Like many toxins identified in ascomycete fungi, its metabolic pathway is dependent on polyketide synthase (PKS) [11], and the other gene functions in the PKS gene clusters have also been determined. However, the biosynthetic pathway of ESCs in *E*. *arachidis* and their potential pathogenic mechanism remain to be explored. For instance, it is unclear whether, in addition to ESCs, there exist cell wall degrading enzymes or effectors that act as virulence factors in *E*. *arachidis* [12].

A growing number of studies have applied genome sequencing technology to the study of phytopathogenic fungi, such as *Magnaporthe oryzae* [13], *Fusarium graminearum* [14], *Sclerotinia sclerotiorum* and *Botrytis cinerea* [15], which has provided new research avenues for a better understanding of their genetic evolution, secondary metabolism, and pathogenic mechanisms.

The present study was aimed at exploring the possible virulence factors of *E*. *arachidis* during host invasion. We report on the 33.18Mb genome sequence of *E*. *arachidis*, the secondary metabolism gene cluster, and the discovery of 6 PKS gene clusters in *E*. *arachidis* including the ESC biosynthetic gene cluster and the core gene *ESCB1*. Through our analysis of the whole genome, we show that *E*. *arachidis* has a complex pathogenesis, with, in addition to the toxin, several candidate virulence factors including effectors, enzymes, and transporters. Moreover, the putative pathogenicity genes provide new horizons to unravel the pathogenic mechanism of *E*. *arachidis*.

## Materials and methods

### Whole-genome sequencing and assembly

In this paper, we used *E*. *arachidis* strain LNFT-H01, which was purified by single spores and cultured on potato dextrose agar (PDA) under 5 microeinstein (μE) m^-2^s^-1^. The genome of LNFT-H01 was sequenced by PacBio RS II using a 20kb library of LNFT-H01 genomic DNA under 100 ×sequencing depth and assembled by Canu [16–18]. The assembled whole-genome sequence, totaling 33.18 Mb and containing 16 scaffolds, was submitted to NCBI (GenBank accession JAAPAX000000000). The characteristics of the genome were mapped in a circus-plot.

### Phylogenetic and syntenic analysis

The evolutionary history can be deduced from conserved sequences and conserved biochemical functions. In addition, clustering the orthologous genes of different genomes can be helpful to integrate the information of conserved gene families and biological processes. We calculated the closest relatives to sequences from *E*. *arachidis* within reference genomes by OrthoMCL, then constructed a phylogenetic tree by SMS implemented in the PhyML (http://www.atgc-montpellier.fr/ phyml-sms/) [19, 20]. Syntenic regions between *E*. *arachidis* and *E*. *australis* were analyzed using MCScanX, which can effectively determine the changes in chromosome structure and reveal the history of the gene family expansion [21].

### Repetitive sequence

Due to the low conservation of repetitive sequence (RS) between species according to MITE Hunter, LTR FINDER, Repeat Scout, and PILER [22–25], we exploited the genome sequence to established a RS database, classified and merged by PASTEClassifier and Repbase [26, 27]. Finally, we predicted the repetitive sequences with RepeatMasker [28].

### Gene prediction and annotation

The ab initio-based and homology-based methods were performed to predict gene numbers in the *E*. *arachidis* genome. A combination of Augustus, Glimmer HMM, Genscan GeneID, and SNAP [29–32] homology-based methods were used by GeMoMa [33] and the results were integrated using EVM [34]. Non-coding RNA including rRNA, tRNA, and other RNAs were also classified and analyzed. According to the structural characteristics of different non-coding RNAs, different strategies were used to predict different non-coding RNAs. Based on the Rfam [35] database, Blastn [36] was used to identify rRNA. We used tRNAscan-SE [37] to identify tRNA. As for the pseudogenes, which have similar sequences to functional genes but have lost their original functions due to mutations, we searched for homologous sequences in the genome through BLAT [38] alignment, and we then used GeneWise [39] to search for immature stop codons and frameshift mutations in the gene sequence to obtain pseudogenes. The preliminary functional annotation was conducted with multiple databases, including the Pfam, NR, KOG/COG, KEGG, and GO databases [40–43]. The pathogen-host interaction (PHI) database, carbohydrate-active enzymes (CAZy) database, and transporter classification database (TCDB) were used to identify potential virulence-related proteins [44–46].

### Identification and characterization of polyketide synthases (PKSs) and secondary metabolite clusters

Secondary metabolite clusters were predicted by performing antiSMASH2 (https://fungismash.Secondarymetabolites.org). In order to confirm the function of polyketide synthase (PKS), which is the core protein that responsible for the biosynthesis of mycotoxin in different organisms, PKS sequences were used to construct the phylogenetic tree by MEGA 10.0.5. The detailed information on PKS is reported in S9 Table. Domains of PKSs were identified via InterPro (https://www.ebi.ac.uk/interpro) and their location visualized by DOG 2.0.

### *ESCB1* expression and toxin determination

Elsinochrome extraction and quantitation were performed as previously described [12]. As for *ESCB1* expression, the strain used for the colony culture was the same as for toxin extraction. Total RNA extraction was done using TransZol^TM^ Up Plus RNA kit (Beijing, TransGen Biotech). RT-PCR was performed using TransScript® One-Step gDNA Removal and cDNA Synthesis (Beijing, TransGen Biotech). qPCR was done using SuperMix TransStart® Green qPCR SuperMix with primers *ESCB1*F (ATCCGAGGTCATTGGTGATG) and *ESCB1*R (GAGGTTGACATCTGGC ATTTG).

## Results

### The characteristics of the whole-genome

**Fig 1:**
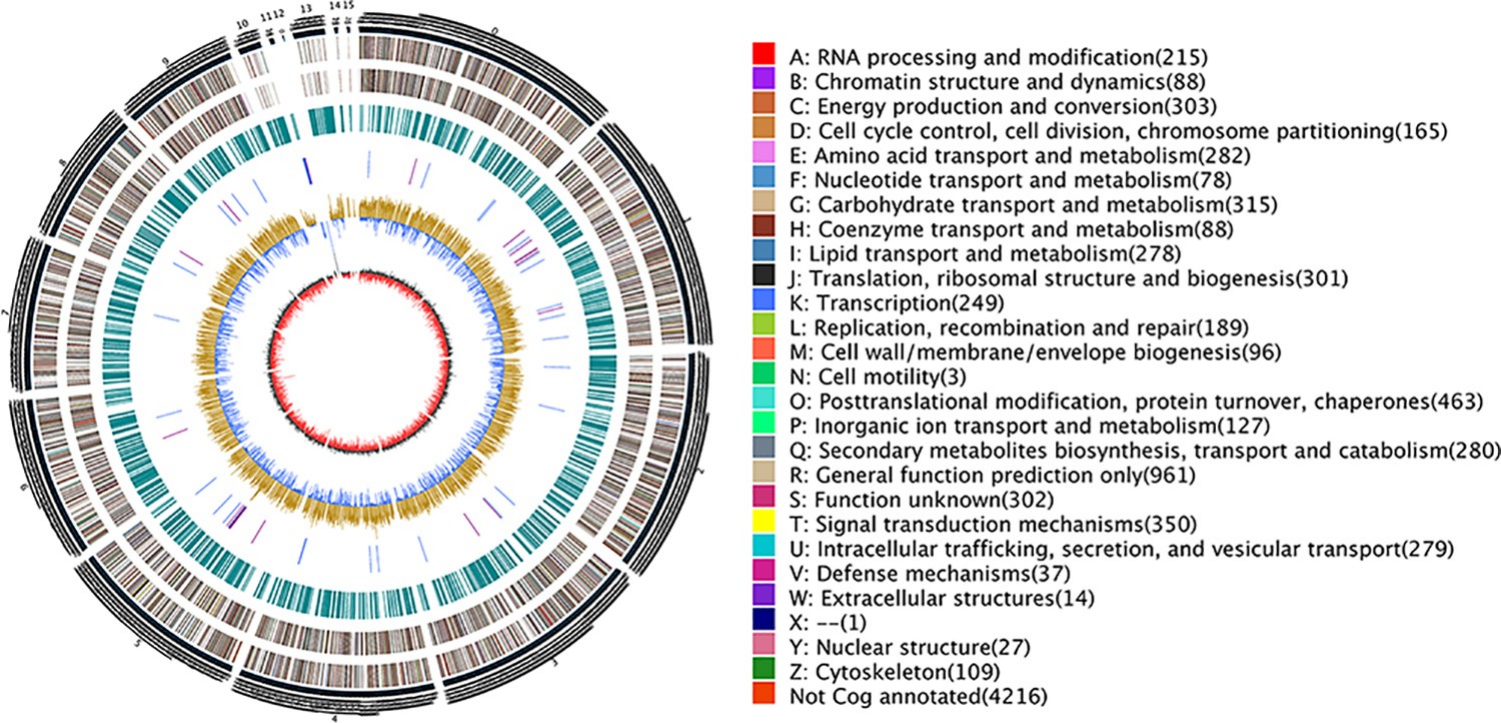
Circos-plot of *E*. *arachidis*. The outermost circle is the size of the genome, each scale is 5 Kb; the second circle and third circle are the genes on the positive and negative strands of the genome, respectively (different colors represent different COG functional); the fourth circle is repeated sequence; the fifth circle is tRNA and rRNA (blue: tRNA, purple: rRNA); the sixth circle is GC content (light yellow: the GC content is higher than the average GC content, blue: the GC content is lower than the average GC content); the innermost circle is GC-skew (dark gray: the G content is greater than C, red: the C content is greater than G).

Whole genome sequencing of *E*. *arachidis* was performed using PacBio RS II (100×coverage). A total of 6.28 Gb high-quality sequencing raw data were assembled by CANU into 16 scaffolds (N50, 3,376,838bp) and the characteristics that are displayed in a circus-plot (Fig 1). We analyzed the genome sequence through Augustus [29] and we identified 7,950 genes. In order to obtain accurate information, we further performed a combination of Glimmer HMM (9,277), Genscan (6,599), GeneID (11,100), and SNAP (10,175) [30–32]. By homology-based methods using GeMoMa [33], taking *E*. *australis* as a reference genome, 8,339 genes were predicted. The above results were integrated by EVM [34] showing that the *E*. *arachidis* genome contains 9,174 genes (Table 1). KOG, KEGG, and GO annotation were in S1 Fig.

**Table 1:** Gene annotation summary statistics.

Genome features	
Genome assembly (Mb)	33.18
Number of coding sequence genes	9,174
GC Content (%)	48.24
PHI	2,752
Secreted protein	734
Transmembrane protein	1,829
TCDB	124

The assembled size of the *E*. *arachidis* genome (33.18 Mb) was comparable in size to the Ascomycota genome (36.91 Mb) [47], as well as *M*. *oryzae*, (38.10 Mb), *Fusarium graminearum* (35.45 Mb), and *Sclerotinia sclerotiorum* (38.68 Mb). However, phylogenetic analysis showed that the species used in this comparative study were distinct from one another. Notably, *E*. *arachidis* was only close to *Sphaceloma murrayae* and *E*. *australis* (S2A Fig), but in terms of genome size, *E*. *arachidis* was larger than *S*. *murrayae* (20.72 Mb) or *E*. *australis* (23.34 Mb). Additionally, synteny analysis indicated the highest synteny between *E*. *arachidis* and *E*. *australis* (S2B Fig). Concerning the identification of repetitive DNA sequences, among 33,184,353bp of the *E*. *arachidis* genome, a total of 7,033,311bp (21.20%) repeat sequences were identified including LTR retrotransposons and DNA transposons (S1 Table).

### Genes associated with detoxification

#### Transporters

**Fig 2:**
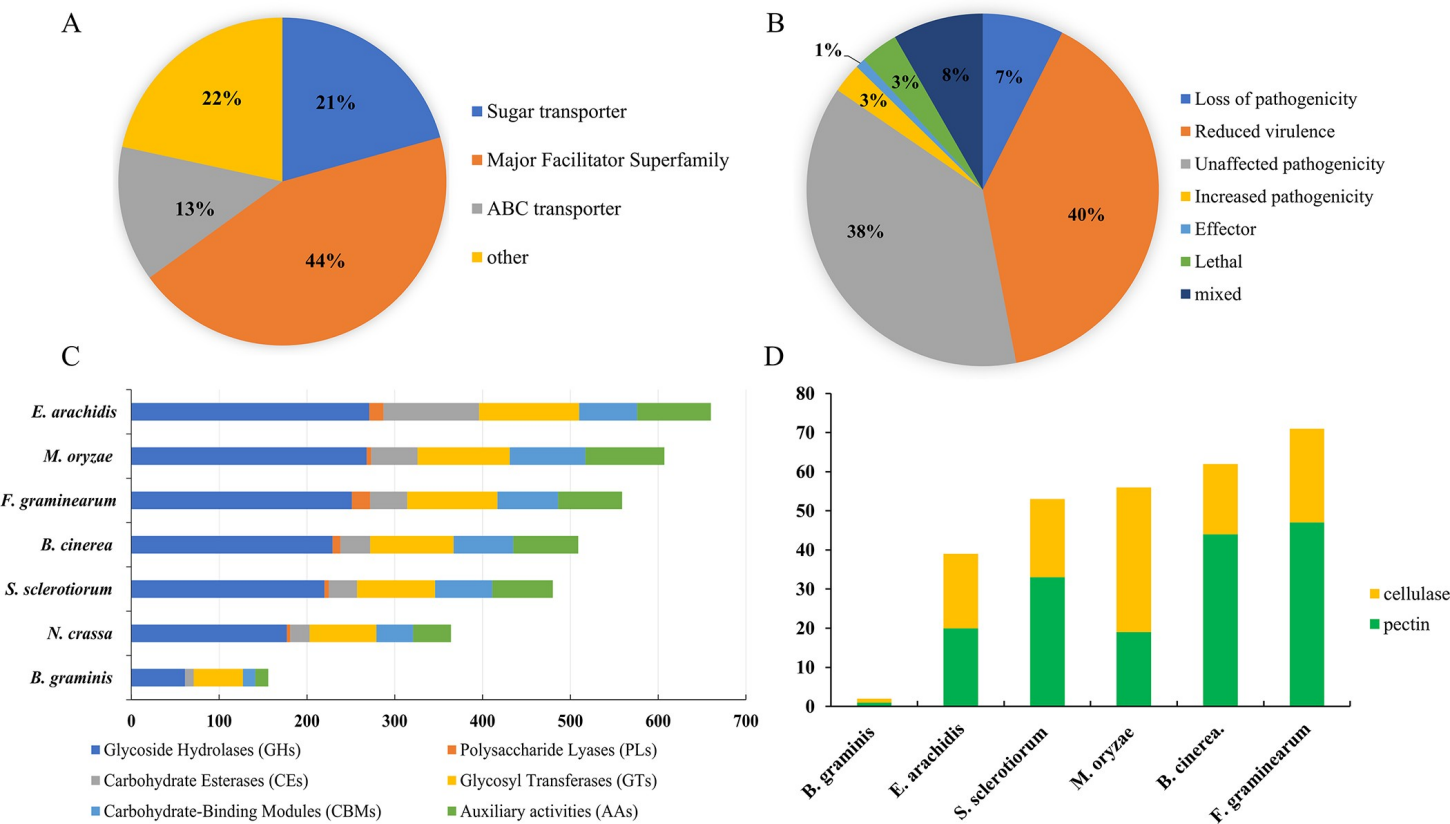
Characteristic of *E*. *arachidis* genome. (A) Transporters of *E*. *arachidis* genome. (B) pathogen-host interaction genes in *E*. *arachidis* genome. (C) CAZymes in compared genomes. (D) Annotation of pectin and cellulase in *E*. *arachidis*.

Transporters are membrane-associated proteins that can assist the movement of ions, amino acids, and macromolecules across the membrane, which plays an important role in a broad range of cellular activities such as nutrient uptake, the release of secondary metabolites, and signal transduction [48]. The major facilitator superfamily (MFS) and ATP-binding cassette (ABC) transporter superfamily are the two largest families of fungal transporters [48]. Among these, the ABC transporters are the primary active transporters, usually as part of multicomponent transporters, that transport different compounds including polysaccharides, heavy metals, oligopeptides, and inorganic ions. In addition, MFS transporters are secondary carriers that facilitate the secretion of endogenous fungal toxins, such as aflatoxins, trichothecenes, and cercosporin. A large number of ABC genes (57) and MFS genes (190) were found in *E*. *arachidis* (S2 Table), which represents 57% of the total number of transporters (Fig 2A). EVM0006810.1, EVM0008188.1, EVM0008646.1, EVM0004073.1, EVM0001603.1, EVM0008241.1, EVM0001951.1, EVM0000776.1, EVM0002663.1 are related to CTB4 (Table 2), which encodes the MFS transporter, and are located in the cercosporin biosynthetic gene cluster. They play a role in the secretion of cercosporin in *Cercospora nicotianae* and are involved in cercosporin resistance [49]. ESC, biosynthesized by *E*. *arachidis*, produces reactive oxygen species in the light acting on the cell membranes and destroying the cellular structure. Meanwhile, *E*. *arachidis* can grow and develop in the presence of high concentrations of reactive oxygen species, which suggesting the certain detoxification of *E*. *arachidis*. The ABC and MFS transporters may play functional roles in the secretion of toxins and play an important role in the virulence toward the plant.

**Table 2:** Detoxification genes in *E*. *arachidis* genome involved in PHI data.

gene ID	PHI annotation	ID	Species
EVM0008224.1	MoCYP51B	G4MZG5	*Magnaporthe oryzae*
EVM0001153.1			
EVM0007235.1	CYP52X1	E2EAF6	*Beauveria bassiana*
EVM0005183.1			
EVM0001975.1			
EVM0006634.1			
EVM0000493.1			
EVM0002264.1			
EVM0004742.1			
EVM0009006.1			
EVM0000504.1			
EVM0001795.1			
EVM0000711.1	cyp51	ABO93363	*Mycosphaerella graminicola*
EVM0007202.1			
EVM0001836.1	CYP51C	I1S2M5	*Fusarium graminearum*
EVM0002479.1			
EVM0006459.1			
EVM0007408.1	CYP1	AAG13968	*Magnaporthe oryzae*
EVM0001986.1			
EVM0006925.1	Cyp51A	I6YDU0	*Fusarium graminearum*
EVM0007146.1	GcABC-G1	F0XP73	*Grosmannia clavigera*
EVM0002882.1			
EVM0000604.1	ABC2	BAC67162	*Magnaporthe oryzae*
EVM0002213.1	ABC3	Q3Y5V5	*Magnaporthe oryzae*
EVM0005164.1			
EVM0004737.1			
EVM0003047.1			
EVM0005246.1			
EVM0005274.1			
EVM0001881.1			
EVM0000747.1			
EVM0001190.1			
EVM0008962.1			
EVM0000397.1			
EVM0003703.1	ABC4	MGG_00937	*Magnaporthe oryzae*
EVM0007439.1			
EVM0002950.1			
EVM0008152.1			
EVM0003958.1			
EVM0000032.1	MgMfs1	A4ZGP3	*Mycosphaerella graminicola*
EVM0007852.1			
EVM0005092.1			
EVM0007310.1			
EVM0003855.1			
EVM0006410.1			
EVM0002601.1			
EVM0005626.1			
EVM0004539.1	BCMFS1	AAF64435	*Botrytis cinerea*
EVM0006582.1			
EVM0002249.1			
EVM0002459.1			
EVM0006810.1	CTB4	A0ST42	*Cercospora nicotianae*
EVM0008188.1			
EVM0008646.1			
EVM0004073.1			
EVM0001603.1			
EVM0008241.1			
EVM0001951.1			
EVM0000776.1			
EVM0002663.1			

#### Cytochrome P450

The cytochrome P450 enzymes (CYPs) are multifunctional oxidoreductases that can aid in the detoxification of natural and environmental pollutants, involved in the primary and secondary metabolism [50]. A total of 78 CYPs (S3 Table) were predicted in *E*. *arachidis* genome, of which 20 CYPs were analyzed in the PHI data (Table 2), mainly including the CYP51 and CYP52 families. The CYP51 families, the conserved fungal P450, are involved in the biosynthesis of membrane ergosterol. MoCYP51B and MoCYP51A both encode a sterol 14α-demethylase enzyme in *M*. *oryzae* that is required for conidiogenesis and mediating the action of sterol demethylation inhibitor (DMI) fungicides [51]. CYP52X1, a member of the CYP52 family, are involved in the degradation of specific epidermal lipid components in the insect waxy layer [52]. In general, the CYPs may be involved in the detoxification of the pathogen’s own toxins.

### Analyses of pathogenicity proteins encoded by the *E*. *arachidis* genome

Through the pathogen-host interaction database, 2,752 potential pathogenic genes were screened in *E*. *arachidis* (Fig 2B), mainly concerning the increased virulence and effectors, the loss of pathogenicity, and reduced virulence as shown in S4 Table.

#### Effectors

During the interaction between pathogens and hosts, pathogens can produce different effector proteins to change the cell structure and metabolic pathways of the host plants, thereby promoting successful infection of the host plants or triggering host defense reactions. In total, 734 genes were predicted to code for secreted proteins in the *E*. *arachidis* genome. Analysis of the PHI database revealed 25 candidate effectors (Table 3) including EVM0006757.1, a gene homologous to PemG1, an elicitor-encoding gene of *Magnaporthe oryzae* which triggered the expression of phenylalanine ammonia-lyase gene [53] and EVM0003806, a gene homologous to glucanase inhibitor protein GPI1 [54] secreted by *Phytophthora sojae*, which inhibits the EGaseA mediated release of elicitor active glucan oligosaccharides from *P*. *sojae* cell wall. The function of candidate effectors from *E*. *arachidis* needs further testing and verification, but also provides a novel research direction for the elucidation of pathogenic mechanisms.

**Table 3:** Effector candidates of *E*. *arachidis* in PHI database.

Effector Candidates	PHI annotation	ID	Species
EVM0000548.1	ANP1	Q6FM27	*Candida glabrata*
EVM0002759.1	Atf1	I1S0C0	*Fusarium graminearum*
EVM0005988.1	ACE1	CAG28797	*Magnaporthe oryzae*
EVM0003884.1	BEC1005	CCU82697	*Blumeria graminis*
EVM0000372.1			
EVM0004193.1			
EVM0007602.1			
EVM0008348.1			
EVM0007402.1	BEC1019	KJ571201	*Blumeria graminis*
EVM0004104.1	BEC1040	CCU82707	*Blumeria graminis*
EVM0002180.1	FRE3	J9VNH2	*Cryptococcus neoformans*
EVM0005699.1			
EVM0000237.1			
EVM0003806.1	GIP1	AAL11720	*Phytophthora sojae*
EVM0003007.1	hopI1	AAL84247	*Pseudomonas syringae*
EVM0006701.1			
EVM0001739.1	mkkA	A0A068BFA5	*Epichloe festucae*
EVM0003220.1	MgSM1	MGG 05344	*Magnaporthe oryzae*
EVM0006757.1	PemG1	ABK56833	*Magnaporthe oryzae*
EVM0001649.1	So (soft)	K9Y567	*Epichloe festucae*
EVM0005038.1			
EVM0005550.1			
EVM0009148.1			
EVM0003400.1	T6SS2	Q6TKU1	*Escherichia coli*
EVM0008814.1			

#### Carbohydrate-active enzymes

The cuticle and cell wall of plants are the primary barriers that prevent the invasion of pathogens. Therefore, the ability to degrade complex plant cell wall carbohydrates such as cellulose and pectin is an indispensable part of the fungal life cycle. The CAZymes secreted by pathogenic fungi are capable of degrading complex plant cell wall carbohydrates to simple monomers that can be used as carbon sources to help pathogen invasion [55]. Mapped *E*. *arachidis* genomes with CAZy database detected 602 genes potentially encoding CAZymes (S6 Table). Subsequently, we compared the CAZyme content to other ascomycetes including necrotrophic plant pathogens (*S*. *sclerotiorum* and *B*. *cinerea*), a biotrophic pathogen (*B*. *graminis*), and hemi-biotrophic pathogens (*M*. *oryzae* and *F*. *graminearum*) (Fig 2C, S7 Table). The CAZyme-content in *E*. *arachidis* is the largest in all compared fungi genomes. This suggests that the CAZymes content does not directly correlate with the lifestyle of the fungus. Further analysis showed, that the pectin and cellulase content of *E*. *arachidis* (39) was smaller than that of the necrotrophic plant pathogens *S*. *sclerotiorum* (53) and *B*. *cinerea* (62). However, it was significantly larger than that of *B*. *graminis* (2) (Fig 2D). In addition to cell wall degrading enzymes, different pathogens likely use different strategies to penetrate plant tissues.

### Secondary metabolism

#### Gene clusters of PKS in *E*. *arachidis*

**Fig 3:**
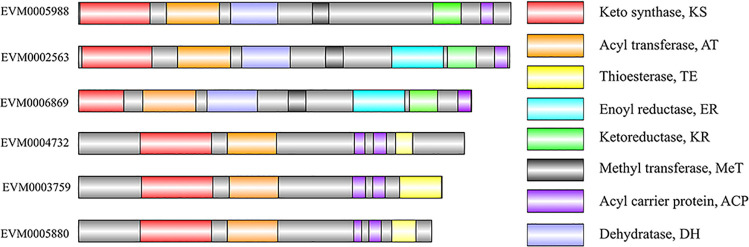
Structure of polyketide synthases proteins. The conservative domain of polyketide synthases was clarified by InterProScan, and the visualization of different domains by using DOG 2.0.

*E*. *arachidis* encodes 24 secondary metabolism clusters, including PKS (6), nonribosomal peptide synthetase (NRPS) (11), NRPS-PKS (1), terpene (6) (S3 Fig). The number of PKS clusters in *E*. *arachidis* were lower than in *M*. *oryzae*, similar to *E*. *fawcettii* and *F*. *graminearum*, but the number of NRPS clusters was twice that of *E*. *fawcettii*, indicating significant differences in metabolic pathways between *E*. *fawcettii* and *E*. *arachidis* (S4 Fig). We analyzed the PKS proteins from *E*. *arachidis* for conserved domains by InterProScan and visualized them using DOG 2.0. (Fig 3). We found that *E*. *arachidis* contains 8 different domains including KS, AT, TE, ER, KR, MeT, ACP, and DH. According to their domain structures, the 6 PKS genes could be further divided into reduced (EVM0002563, EVM0005988, EVM0006869) and non-reduced (EVM0003759, EVM0004732, EVM0005880) due to the reducing activity of ER and KR.

**Fig 4:**
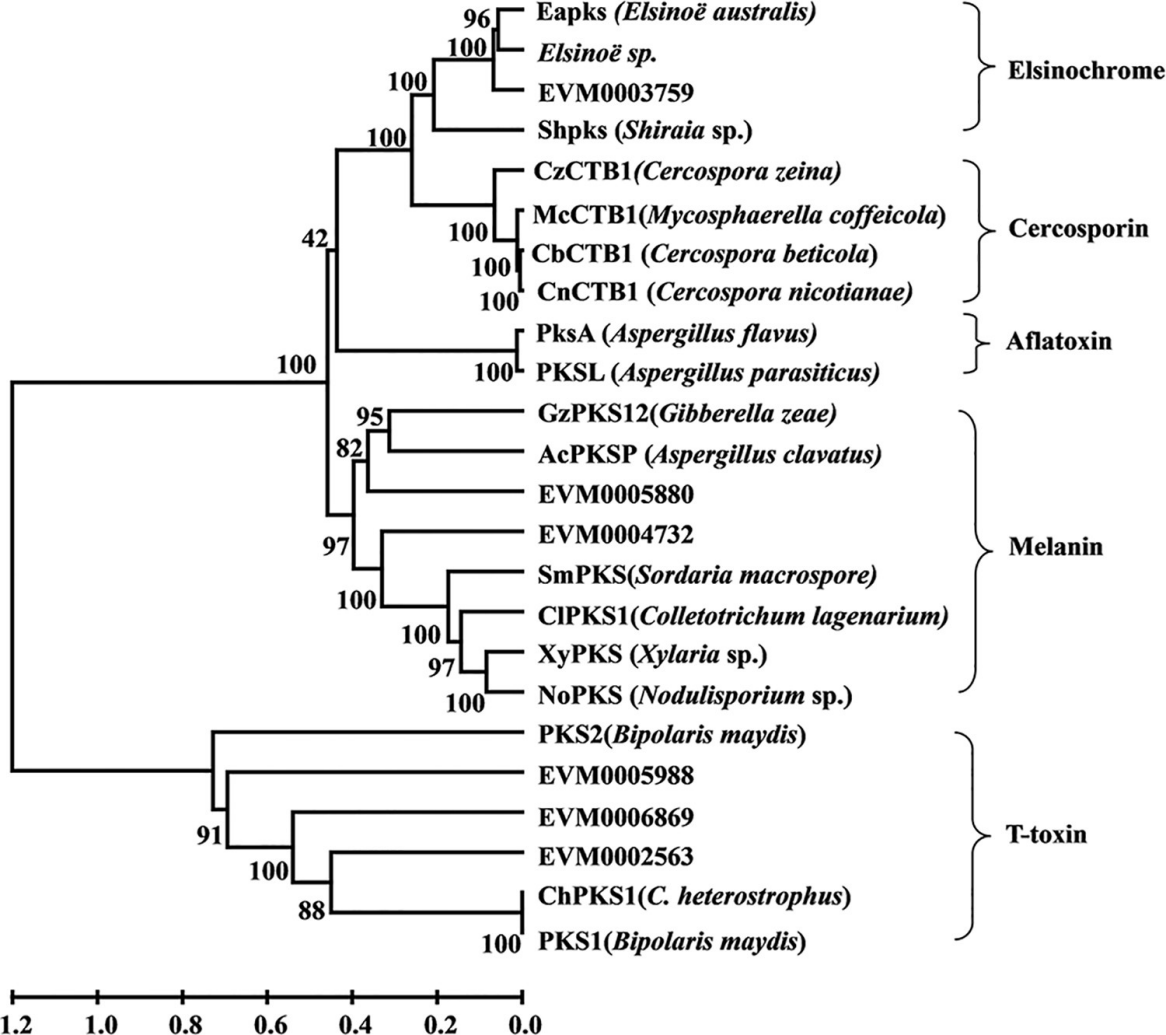
Phylogenetic analyses of *E*. *arachidis* and other fungal polyketide synthases (PKS). Phylogenetic tree was constructed with PKS sequences from different organisms which classified with the types of reducing domains are divided into five clades.

In order to further differentiate the 6 PKS genes, 19 different PKS genes were analyzed (S8 Table). Among the 6 PKS from *E*. *arachidis*, EVM0003759 was in the same clade as EaPKS which is encoding for ESC biosynthesis in *E*. *australis* and therefore we named it ESCB1 (**E**l**s**ino**c**hrome **B**iosynthesis gene 1). Interestingly, EVM0004732 and EVM0005880 are related to the biosynthesis of melanin (Fig 4). This is the first time that melanin has been predicted in this pathogen. Whether melanin in *E*. *arachidis* plays a role in pathogenicity as it does in *M*. *oryzae* by aiding to penetrate the host plant remains to be verified.

#### Expression of ESCB1 and analysis of flanking genes in *E*. *arachidis*

**Fig 5:**
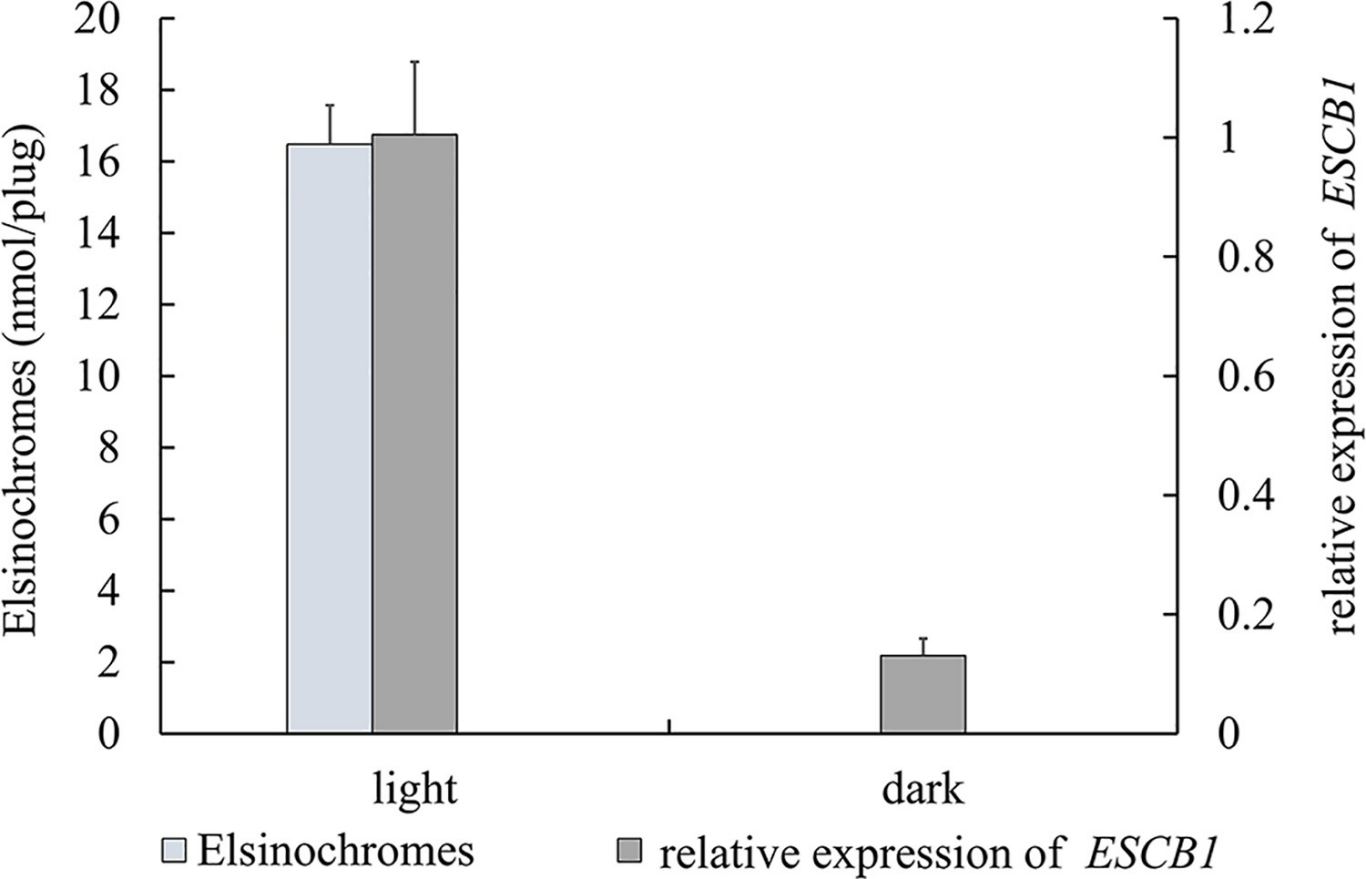
ESC and expression levels analysis of *ESCB1*. ESC and expression levels of *ESCB1* was investigated in light and dark condition, respectively.

**Fig 6:**
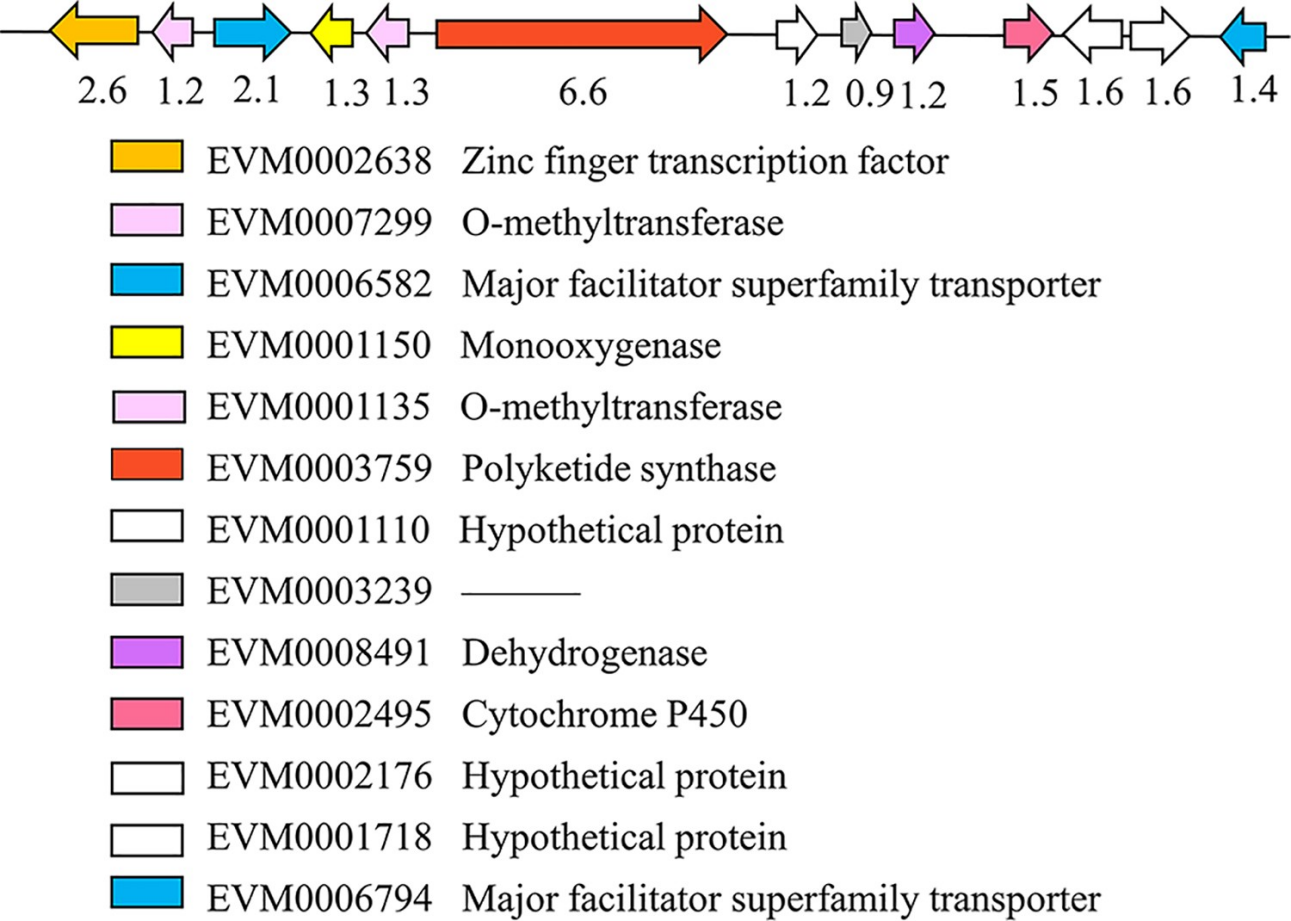
Distribution of the *ESCB1* gene cluster. BLASTX was used to search the NCBI database to predict the function of related genes.

Noteworthily, we previously determined that the content of ESC in *E*. *arachidis* was obviously decreased under dark conditions [12]. We compared the toxin content and *ESCB1* expression under light and dark conditions, as expected, the change tendency was similarity (Fig 5). 13 putative Open Reading Frames were identified in the flanking of *ESCB1* (Fig 6), including EVM0001135 and EVM0007299 which encode O-methyltransferase, EVM0006582 and EVM0006794 similarity to MFS transporter, EVM0002495 Cytochrome P450, and EVM0002638 zinc finger transcription factor.

## Discussion

*Elsinoë* species cause scab and spot anthracnose on various crops including peanut, cassava, citrus, mango, and grape. In this paper, the first whole genome sequence of *E*. *arachidis* were reported and revealed the complex gene structures that may be involved in its pathogenic mechanism. Additionally, we predicted the ESC toxin biosynthesis gene cluster. The genome size of *E*. *arachidis* is 33.18Mb, which was comparable in size to the Ascomycota genome size, however, compared with *E*. *australis* (23.34 Mb), *E*. *arachidis* has a larger genome size. This may be due to the lower proportion of repeat sequences in the *E*. *fawcettii* genome [56]. The GC content was 48.24% and CDSs percentage of the genome was 43.94%.

Mycotoxins play an important part in the pathogenic mechanisms of pathogens. Mycotoxin ESCs, perylenequinones photosensitive toxins, can produce reactive oxygen species (ROS) and act on the cell membrane to destroy the cell structure. *E*. *arachidis* can maintain growth and development even in the presence of high toxin levels, which indicates an efficient self-detoxification mechanism. We identified ABC transporters and MFS transporters in *E*. *arachidis* indicating the complex transportation of substances in *E*. *arachidis* and that some of them may have an effect on the secretion of ESCs. Cytochrome P450 enzyme system, a multifunctional oxidoreductase, may involve in the self-detoxification of *E*. *arachidis* by providing redox conditions to maintain its own steady state for various physiological and biochemical reactions.

ESC is a crucial virulent factor in the pathogenic process of *E*. *arachidis*. However, compared with mycotoxins such as aflatoxins, fumonisin, and trichothecenes, and host-selective toxins such as T-toxin, still little is known about the biosynthetic pathways of perylenequinone mycotoxins. Cercosporin, the same group of perylenequinone toxins with ESC, has been proved that *CTB1* (cercosporin synthase gene 1) which encoding polyketide synthase is the core gene of cercosporin biosynthesis pathway [10]. *Efpks1* has been shown to function the in ESC biosynthesis in *E*. *fawcettii*, but the specific biosynthesis pathway still needs to be further clarified [8, 9]. With the prediction of the secondary metabolism gene cluster of *E*. *arachidis*, 6 gene clusters related to polyketide synthase were obtained. The core genes were *EVM0002563*, *EVM0003759*, *EVM0004732*, *EVM0005880*, *EVM0005988*, and *EVM0006869*. Phylogenetic tree constructions showed that EVM0003759 is involved in ESCs synthesis, while EVM0004732 and EVM0005880 play a role in melanin synthesis. To our knowledge, this is the first time that melanin has been identified in *E*. *arachidis*. Interestingly, analysis of the position between the core genes of ESCs and melanin gene clusters, we found that the three genes are all located in Contig00003. This result also cast some doubt on whether PKS synthesis pathways from ESC and melanin are interrelated or competing.

Pathogens employ complex mechanisms to break through the defenses of plants, including toxins, enzymes, and other pathogenic factors to help invasion and colonization. Analysis of the CAZy and PHI databases revealed that, in addition to ESCs, enzymes, effectors, and certain transcription factors may be involved in the pathogenic process. Increased virulence factors (3%) that cause increased pathogenicity include O-methylsterigmatocystin oxidoreductase, AK-toxin biosynthetic gene 7 (AKT7) and bZIP transcription factor MeaB. EVM0005728, EVM0001699 and EVM0004784 are related to AKT7, which encodes a cytochrome P450 monooxygenase in *Alternaria alternata* and can limit the host-selective toxin AK-toxin production [57]. EVM0002472 is endowed with a basic leucine zipper (bZIP) domain similar to the MeaB transcription factor in *Fusarium oxysporum* [58], which activates a conserved nitrogen responsive pathway to control the virulence of plant pathogenic fungi (S5 Table).

In conclusion, we reported the whole-genome sequence of *E*. *arachidis*. Analysis of its assembly and annotation allowed the identification of the presumptive PKS gene clusters. Based on our results, we hypothesize that *ESCB1* maybe the core gene of the biosynthesis of ESC. Additionally, pathogenic factors including CAZymes and effectors may help *E*. *arachidis* to circumvent the defense mechanisms of peanuts. Our work lays the foundation of future research aimed at elucidating the detailed pathogenic mechanisms of *E*. *arachidis*.

## Conclusions

In conclusion, this is the first report of the high-quality genome of *E*. *arachidis* by PacBio RS II. The basic information of the sequence, gene family and metabolic gene cluster of *E*. *arachidis* were clarified. Through further analysis of the key genes in different PKS gene clusters, the expression of *ESCB1* (EVM0003759) under light and dark condition was initially determined to participate in the ESC biosynthetic pathway, and the flanking sequences of this gene cluster were annotation, including major facilitator superfamily transporter, cytochrome P450, monooxygenase and O-methyltransferase. In addition to ESC toxins, genes related to mycotoxin biosynthesis such as melanin are also noted. This information provides new ideas for further exploration of the pathogenic mechanism of *E*. *arachidis*.

## Supporting information

S1 Fig GO, KOG and KEGG annotation of *E. arachidis*. (TIF)

S2 Fig Collinear analysis and evolutionary analysis of *E. arachidis*. (A) A phylogenetic tree constructed the evolutionary relationships of *E*. *arachidis* and other fungi. (B) Collinear analysis.
(TIF)

S3 Fig Gene clusters in *E. arachidis*. (TIF)

S4 Fig PKS, NRPS and NRPS-PKS hybrid in different genome. (TIF)

S1 Table Repetitive sequence in *E. arachidis*. (DOC)

S2 Table ABC transporter and major facilitator superfamily in *E. arachidis*. (XLSX)

S3 Table Cytochrome P450 in *E. arachidis*. (XLSX)

S4 Table The loss of pathogenicity and reduced virulence genes in *E. arachidis*. (DOCX)

S5 Table Increased virulence genes in *E. arachidis*. (DOCX)

S6 Table CAZyme_family in *E. arachidis*. (XLSX)

S7 Table CAZymes in *E. arachidis* and compared genome. (DOCX)

S8 Table The information of the different PKSs. (DOC)

## References

[1] Zhou R J, Xu Z, Fu J F, Cui J C, He J J, Xue C Y. Resistance evaluation of peanut varieties to peanut scab and the epidemic dynamics in Liaoning Province. Acta Phytophylacica Sinica. 2014;41(5):597–601. doi: 10.13802/j.cnki.zwbhxb.2014.05.033

[2] Fang S M, Wang Z R, Ke Y Q, Chen Y S, Huang C M, Yu J X. The Evaluation of Resistance and Resistant Mechanisms of Peanut Varieties to Scab Disease. Scientia Agricultura Sinica. 2007; 40(2):291–297. doi: 10.3321/j.issn:0578–1752.2007.02.011

[3] Fan X L, Barreto R W, Groenewald J Z, Bezerra J D P, Pereira O L, Cheewangkoon R, et al. Phylogeny and taxonomy of the scab and spot anthracnose fungus *Elsinoë* (Myriangiales, Dothideomycetes). Studies in Mycology. 2017; 87(C):1–41. doi: 10.1016/j.simyco.2017.02.001 28373739 PMC5367849

[4] Zhao J F, Zhou R J, Li Y J, Lu L, Fu J F, Xue C Y. Infectious condition of *Sphaceloma arachidis* and physiological responses to pathogen infection in peanut. Chinese Journal of Oil Crop Sciences. 2017; (1) doi: 10.7505/j.issn.1007-9084.2017.01.015

[5] Lu Liu, Wenli Jiao, Rujun Zhou, Yuanjie Li, Mengxue Xu, Junfan Fu. Extraction Technology and Activity Analysis of *Elsinoë arachidis* Toxin. Journal of Shenyang Agricultural University. 2018; 49(3): 272–278. doi: 10.3969/j.issn.1000-1700.2018.03.003

[6] Fang SM, Wang ZR, Guo JM. Fungicides selection for peanut scab disease, Chinese Journal of Oil Crop Sciences. 2006; 28, 220–223.

[7] Weiss U, Flon H, Burger WC. The photodynamic pigment of some species of *Elsinoë* and *Sphaceloma*. *Arch Biochem Biophys*. 1957;69:311–319. doi: 10.1016/0003-9861(57)90497-6 13445204

[8] Liao HL, Chung KR. Cellular toxicity of elsinochrome phytotoxins produced by the pathogenic fungus, *Elsinoë fawcettii* causing citrus scab. *New Phytol*. 2008;177(1):239–250. doi: 10.1111/j.1469-8137.2007.02234.x 17953652

[9] Daub ME, Herrero S, Chung KR. Photoactivated perylenequinone toxins in fungal pathogenesis of plants. *FEMS Microbiol Lett*. 2005;252(2):197–206. doi: 10.1016/j.femsle.2005.08.033 16165316

[10] Choquer M, Dekkers KL, Chen HQ, et al. The CTB1 gene encoding a fungal polyketide synthase is required for cercosporin biosynthesis and fungal virulence of *Cercospora nicotianae*. *Mol Plant Microbe Interact*. 2005;18(5):468–476. doi: 10.1094/MPMI-18-0468 15915645

[11] Daub ME. Cercosporin, a photosensitizing toxin from *Cercospora* species. Phytopathology. 1982; 72, 370–374. doi: 10.1094/Phyto-77-370

[12] Jiao W, Liu L, Zhou R, Xu M, Xiao D, Xue C. Elsinochrome phytotoxin production and pathogenicity of Elsinoë arachidis isolates in China. *PLoS One*. 2019;14(6):e0218391. doi: 10.1371/journal.pone.0218391 31194853 PMC6564019

[13] Dong Y, Li Y, Zhao M, et al. Global genome and transcriptome analyses of Magnaporthe oryzae epidemic isolate 98–06 uncover novel effectors and pathogenicity-related genes, revealing gene gain and lose dynamics in genome evolution. PLoS Pathog. 2015;11(4):e1004801. doi: 10.1371/journal.ppat.1004801 25837042 PMC4383609

[14] Walkowiak S, Rowland O, Rodrigue N, Subramaniam R. Whole genome sequencing and comparative genomics of closely related *Fusarium* Head Blight fungi: *Fusarium graminearum*, *F*. *meridionale* and *F*. *asiaticum*. *BMC Genomics*. 2016;17(1):1014. doi: 10.1186/s12864-016-3371-1 27938326 PMC5148886

[15] Amselem J, Cuomo C A, van Kan JA, et al., Genomic Analysis of the Necrotrophic Fungal Pathogens *Sclerotinia sclerotiorum* and *Botrytis cinerea*. *PLoS Genetics*. 2011, 7(8):e1002230. doi: 10.1371/journal.pgen.1002230 21876677 PMC3158057

[16] Chin CS, Alexander DH, Marks P, et al. Nonhybrid, finished microbial genome assemblies from long-read SMRT sequencing data. *Nat Methods*. 2013;10(6):563–569. doi: 10.1038/nmeth.2474 23644548

[17] Berlin K, Koren S, Chin C S, Drake J P, Landolin J M, and Phillippy A M. Assembling large genomes with single-molecule sequencing and locality-sensitive hashing. *Nature biotechnology*. 2015; 33, 623–630 doi: 10.1038/nbt.3238 26006009

[18] Koren S, Walenz BP, Berlin K, Miller JR, Bergman NH, Phillippy AM. Canu: scalable and accurate long-read assembly via adaptive *k*-mer weighting and repeat separation. *Genome Res*. 2017;27(5):722–736. doi: 10.1101/gr.215087.116 28298431 PMC5411767

[19] Lefort V, Longueville JE, Gascuel O. SMS: Smart Model Selection in PhyML. *Mol Biol Evol*. 2017;34(9):2422–2424. doi: 10.1093/molbev/msx149 28472384 PMC5850602

[20] Li L, Stoeckert CJ Jr, Roos DS. OrthoMCL: identification of ortholog groups for eukaryotic genomes. *Genome Res*. 2003;13(9):2178–2189. doi: 10.1101/gr.1224503 12952885 PMC403725

[21] Wang Y, Tang H, Debarry JD, et al. MCScanX: a toolkit for detection and evolutionary analysis of gene synteny and collinearity. *Nucleic Acids Res*. 2012;40(7):e49. doi: 10.1093/nar/gkr1293 22217600 PMC3326336

[22] Xu Z, Wang H. LTR_FINDER: an efficient tool for the prediction of full-length LTR retrotransposons. *Nucleic Acids Res*. 2007;35(Web Server issue):W265–W268. doi: 10.1093/nar/gkm286 17485477 PMC1933203

[23] Han Y, Wessler SR. MITE-Hunter: a program for discovering miniature inverted-repeat transposable elements from genomic sequences. *Nucleic Acids Res*. 2010;38(22):e199. doi: 10.1093/nar/gkq862 20880995 PMC3001096

[24] Price AL, Jones NC, Pevzner PA. De novo identification of repeat families in large genomes. Bioinformatics. 2005; 21, 351–358. doi: 10.1093/bioinformatics/bti1018 15961478

[25] Edgar RC, Myers EW. PILER: identification and classification of genomic repeats. *Bioinformatics*. 2005;21 Suppl 1:i152–i158 doi: 10.1093/bioinformatics/bti1003 15961452

[26] Wicker T, Sabot F, Hua-Van A, et al. A unified classification system for eukaryotic transposable elements. *Nat Rev Genet*. 2007;8(12):973–982. doi: 10.1038/nrg2165 17984973

[27] Jurka J, Kapitonov VV, Pavlicek A, Klonowski P, Kohany O, Walichiewicz J. Repbase Update, a database of eukaryotic repetitive elements. *Cytogenet Genome Res*. 2005;110(1–4):462–467. doi: 10.1159/000084979 16093699

[28] Tarailo-Graovac M, Chen N. Using RepeatMasker to identify repetitive elements in genomic sequences. *Curr Protoc Bioinformatics*. 2009;Chapter 4. doi: 10.1002/0471250953.bi0410s25 19274634

[29] Stanke M, Waack S. Gene prediction with a hidden Markov model and a new intron submodel. *Bioinformatics*. 2003; 19, 215–225. doi: 10.1093/bioinformatics/btg1080 14534192

[30] Blanco E, Parra G, Guigó R. Using geneid to identify genes. *Curr Protoc Bioinformatics*. 2007;Chapter 4. doi: 10.1002/0471250953.bi0403s18 18428791

[31] Majoros WH, Pertea M, Salzberg SL. TigrScan and GlimmerHMM: two open source ab initio eukaryotic gene-finders. *Bioinformatics*. 2004;20(16):2878–2879. doi: 10.1093/bioinformatics/bth315 15145805

[32] Korf I. Gene finding in novel genomes. *BMC Bioinformatics*. 2004;5:59. doi: 10.1186/1471-2105-5-59 15144565 PMC421630

[33] Keilwagen J, Hartung F, Grau J. GeMoMa: Homology-Based Gene Prediction Utilizing Intron Position Conservation and RNA-seq Data. *Methods Mol Biol*. 2019;1962:161–177 doi: 10.1007/978-1-4939-9173-0_9 31020559

[34] Haas BJ, Salzberg SL, Zhu W, et al. Automated eukaryotic gene structure annotation using EVidenceModeler and the Program to Assemble Spliced Alignments. *Genome Biol*. 2008;9(1):R7. doi: 10.1186/gb-2008-9-1-r7 18190707 PMC2395244

[35] Griffiths-Jones S, Moxon S, Marshall M, Khanna A, Eddy SR, Bateman A. Rfam: annotating non-coding RNAs in complete genomes. *Nucleic Acids Res*. 2005;33(Database issue):D121–D124. doi: 10.1093/nar/gki081 15608160 PMC540035

[36] Altschul SF, Gish W, Miller W, Myers EW, Lipman DJ: Basic local alignment search tool. *Journal of molecular biology*. 1990, 215:403–410. doi: 10.1016/S0022-2836(05)80360-2 2231712

[37] Lowe TM, Eddy SR. tRNAscan-SE: a program for improved detection of transfer RNA genes in genomic sequence. *Nucleic Acids Res*. 1997;25(5):955–964. doi: 10.1093/nar/25.5.955 9023104 PMC146525

[38] Kent WJ. BLAT—the BLAST-like alignment tool. *Genome research* 2002, 12:656–664. doi: 10.1101/gr.229202 11932250 PMC187518

[39] Birney E, Clamp M, Durbin R. GeneWise and genomewise. Genome research. 2004; 14:988–995. doi: 10.1101/gr.1865504 15123596 PMC479130

[40] Finn RD, Bateman A, Clements J, et al. Pfam: the protein families database. *Nucleic Acids Res*. 2014;42(Database issue):D222–D230. doi: 10.1093/nar/gkt1223 24288371 PMC3965110

[41] Tatusov RL, Galperin MY, Natale DA, Koonin EV. The COG database: a tool for genome-scale analysis of protein functions and evolution. *Nucleic Acids Res*. 2000;28(1):33–36. doi: 10.1093/nar/28.1.33 10592175 PMC102395

[42] Kanehisa M, Goto S, Kawashima S, Okuno Y, Hattori M. The KEGG resource for deciphering the genome. *Nucleic Acids Res*. 2004;32(Database issue):D277–D280. doi: 10.1093/nar/gkh063 14681412 PMC308797

[43] Ashburner M, Ball C A, Blake J A. Gene Ontology: tool for the unification of biology. Nature genetics. 2000; 25, 25–29. doi: 10.1038/75556 10802651 PMC3037419

[44] Lombard V, Golaconda Ramulu H, Drula E, Coutinho PM, Henrissat B. The carbohydrate-active enzymes database (CAZy) in 2013. *Nucleic Acids Res*. 2014;42(Database issue):D490–D495. doi: 10.1093/nar/gkt1178 24270786 PMC3965031

[45] Saier MH Jr, Tran CV, Barabote RD. TCDB: The Transporter Classification Database for membrane transport protein analyses and information. *Nucleic Acids Res*. 2006;34(Database issue):D181–D186. doi: 10.1093/nar/gkj001 16381841 PMC1334385

[46] Winnenburg R, Baldwin TK, Urban M, Rawlings C, Köhler J, Hammond-Kosack KE. PHI-base: a new database for pathogen host interactions. *Nucleic Acids Res*. 2006;34(Database issue):D459–D464. doi: 10.1093/nar/gkj047 16381911 PMC1347410

[47] Mohanta TK, Bae H. The diversity of fungal genome. *Biol Proced Online*. 2015;17:8. doi: 10.1186/s12575-015-0020-z 25866485 PMC4392786

[48] Perlin MH, Andrews J, Toh SS. Essential letters in the fungal alphabet: ABC and MFS transporters and their roles in survival and pathogenicity. *Adv Genet*. 2014;85:201–253. doi: 10.1016/B978-0-12-800271-1.00004-4 24880736

[49] Choquer M, Lee MH, Bau HJ, Chung KR. Deletion of a MFS transporter-like gene in *Cercospora nicotianae* reduces cercosporin toxin accumulation and fungal virulence. *FEBS Lett*. 2007;581(3):489–494. doi: 10.1016/j.febslet.2007.01.011 17250832

[50] Guengerich FP. Cytochrome P450 research and *The Journal of Biological Chemistry*. *J Biol Chem*. 2019;294(5):1671–1680. doi: 10.1074/jbc.TM118.004144 29871932 PMC6364786

[51] Yan X, Ma WB, Li Y, et al. A sterol 14α-demethylase is required for conidiation, virulence and for mediating sensitivity to sterol demethylation inhibitors by the rice blast fungus *Magnaporthe oryzae*. *Fungal Genet Biol*. 2011;48(2):144–153. doi: 10.1016/j.fgb.2010.09.005 20887796

[52] Pedrini N, Ortiz-Urquiza A, Huarte-Bonnet C, Zhang S, Keyhani NO. Targeting of insect epicuticular lipids by the entomopathogenic fungus *Beauveria bassiana*: hydrocarbon oxidation within the context of a host-pathogen interaction. *Front Microbiol*. 2013;4:24. doi: 10.3389/fmicb.2013.00024 23422735 PMC3573267

[53] Qiu D, Mao J, Yang X, Zeng H. Expression of an elicitor-encoding gene from *Magnaporthe grisea* enhances resistance against blast disease in transgenic rice. *Plant Cell Rep*. 2009;28(6):925–933. doi: 10.1007/s00299-009-0698-y 19337737

[54] Rose JK, Ham KS, Darvill AG, Albersheim P. Molecular cloning and characterization of glucanase inhibitor proteins: coevolution of a counterdefense mechanism by plant pathogens. *Plant Cell*. 2002;14(6):1329–1345. doi: 10.1105/tpc.002253 12084830 PMC150783

[55] van den Brink J, de Vries RP. Fungal enzyme sets for plant polysaccharide degradation. *Appl Microbiol Biotechnol*. 2011;91(6):1477–1492. doi: 10.1007/s00253-011-3473-2 21785931 PMC3160556

[56] Jeffress S, Arun-Chinnappa K, Stodart B, Vaghefi N, Tan YP, Ash G. Genome mining of the citrus pathogen *Elsinoë fawcettii*; prediction and prioritisation of candidate effectors, cell wall degrading enzymes and secondary metabolite gene clusters. PLoS One. 2020;15(5):e0227396. doi: 10.1371/journal.pone.0227396 32469865 PMC7259788

[57] Takaoka S, Kurata M, Harimoto Y, et al. Complex regulation of secondary metabolism controlling pathogenicity in the phytopathogenic fungus *Alternaria alternata*. *New Phytol*. 2014;202(4):1297–1309. doi: 10.1111/nph.12754 24611558

[58] López-Berges MS, Rispail N, Prados-Rosales RC, Di Pietro A. A nitrogen response pathway regulates virulence functions in *Fusarium oxysporum* via the protein kinase TOR and the bZIP protein MeaB. *Plant Cell*. 2010, 22(7):2459–75. doi: 10.1105/tpc.110.075937 20639450 PMC2929112

