# Supplementary figures and images for: Genomic analysis of Elsinoë arachidis reveals its potential pathogenic mechanism and the biosynthesis pathway of elsinochrome toxin
Source: PLoS One. 2021 Dec 16;16(12):e0261487. doi: 10.1371/journal.pone.0261487 (PMC8675698; doi:10.1371/journal.pone.0261487)

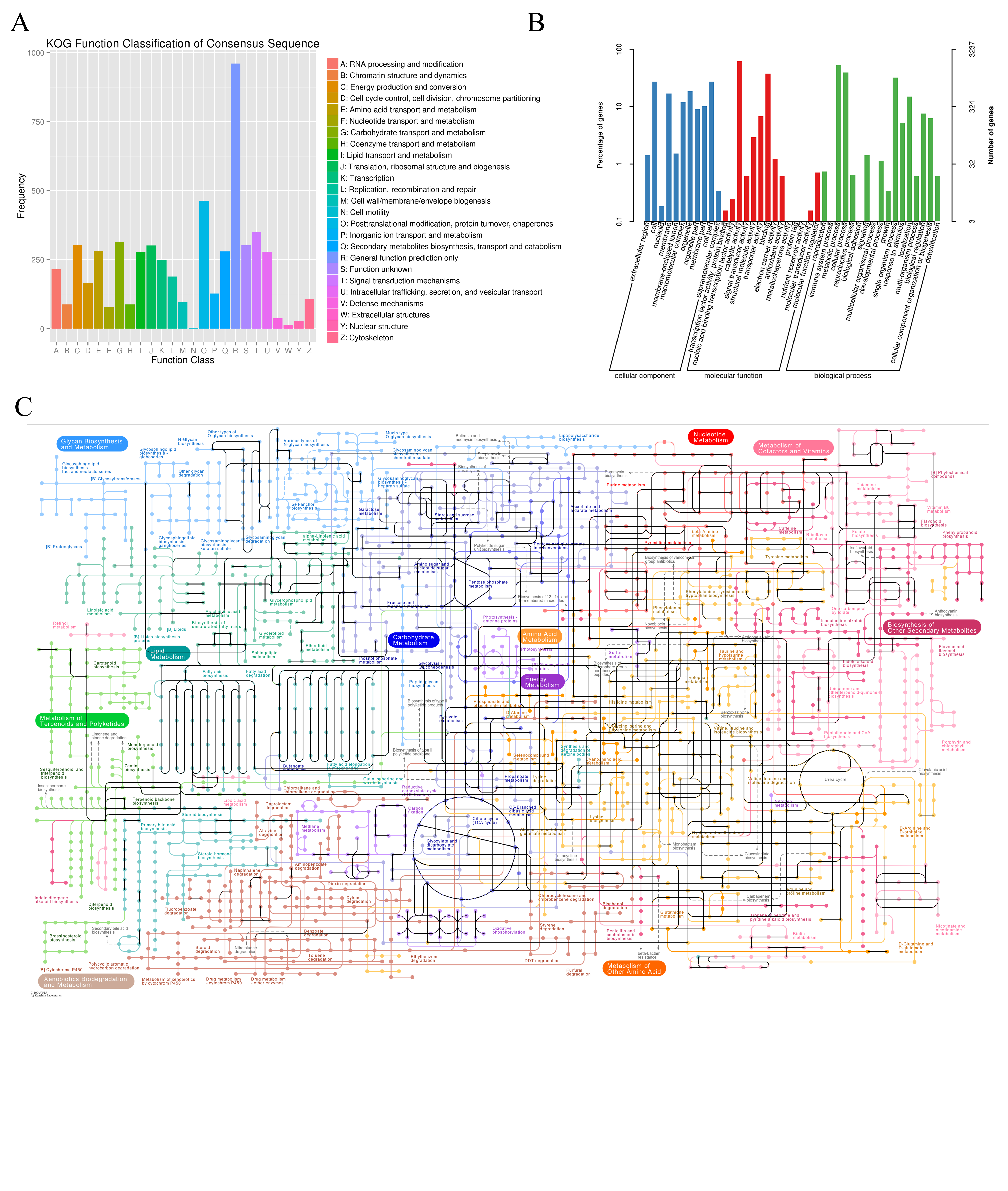

Supplement: S1 Fig — (TIF) [file pone.0261487.s001.tif]

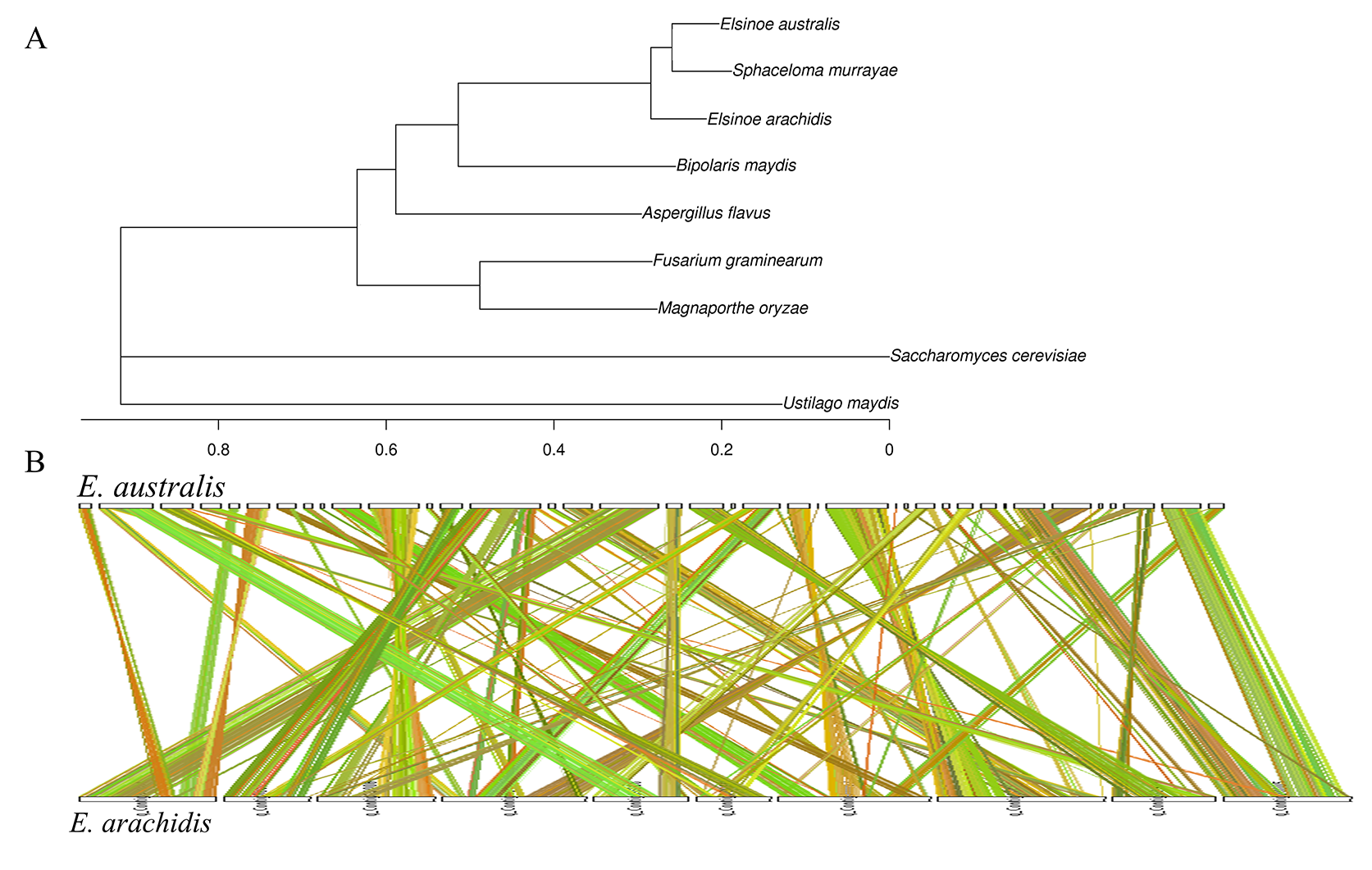

Supplement: S2 Fig — (A) A phylogenetic tree constructed the evolutionary relationships of E. arachidis and other fungi. (B) Collinear analysis. (TIF) [file pone.0261487.s002.tif]

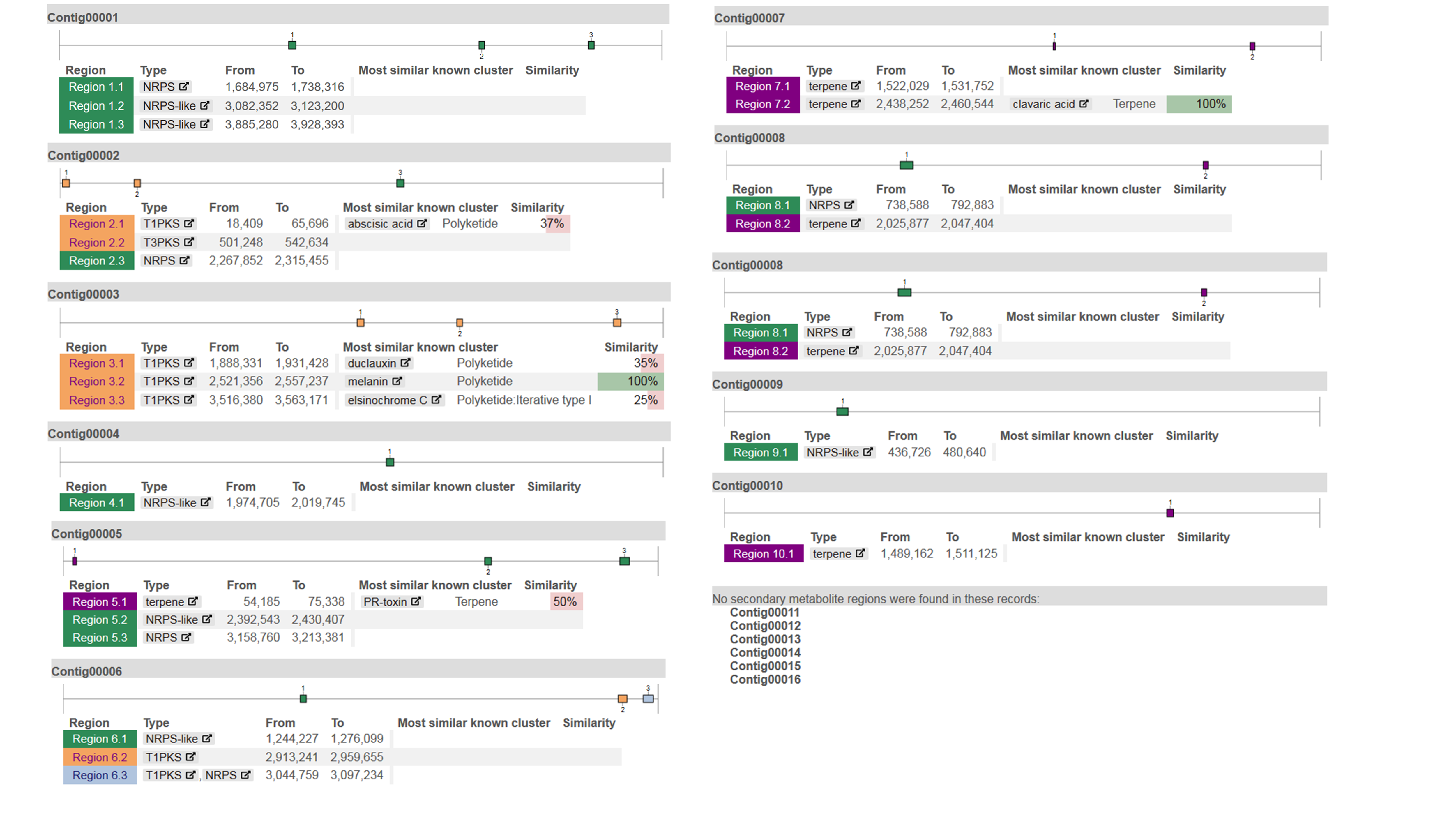

Supplement: S3 Fig — (TIF) [file pone.0261487.s003.tif]

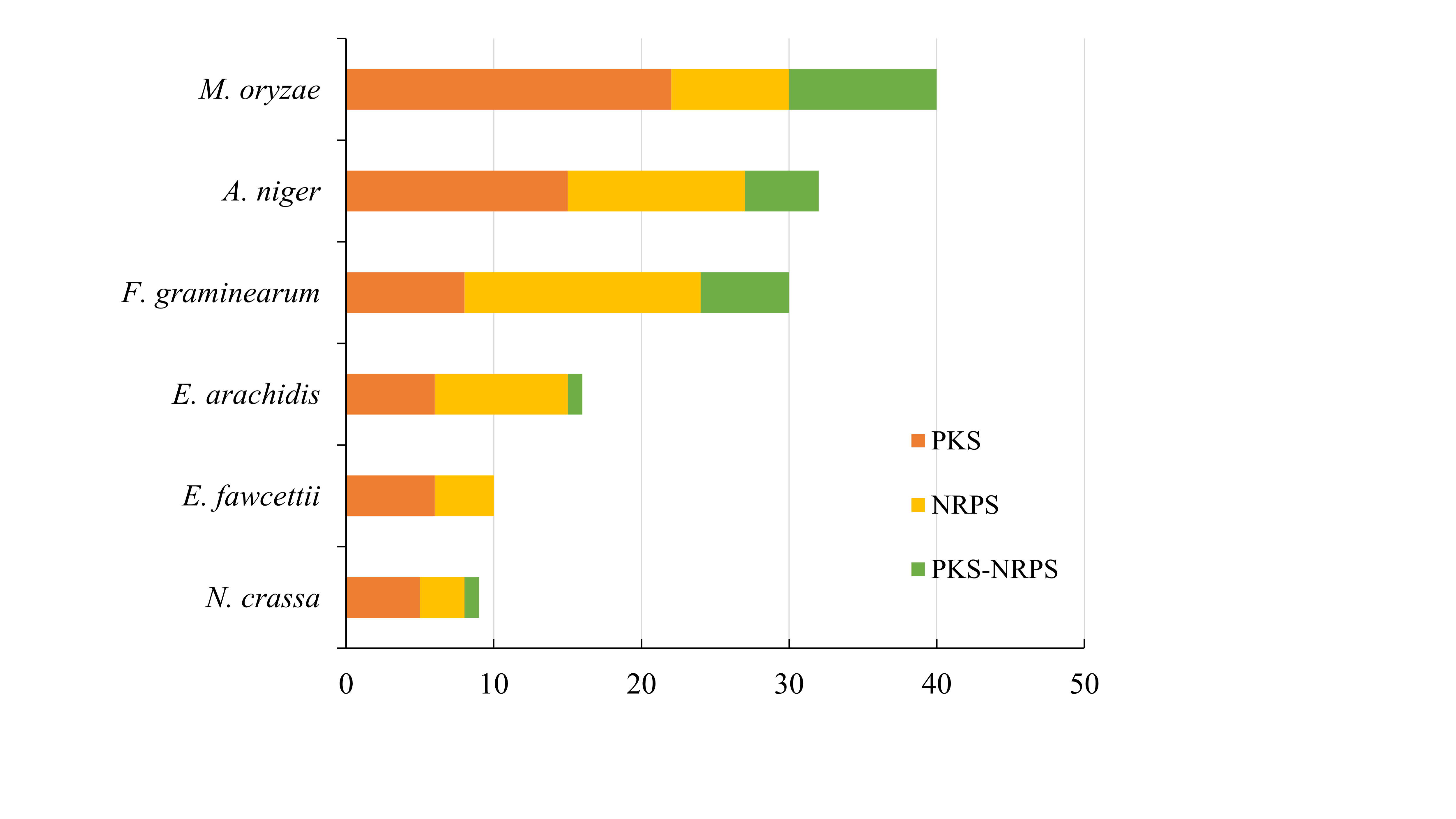

Supplement: S4 Fig — (TIF) [file pone.0261487.s004.tif]
