# Supplementary material for: Genomic analysis of Elsinoë arachidis reveals its potential pathogenic mechanism and the biosynthesis pathway of elsinochrome toxin
Source: PLoS One. 2021 Dec 16;16(12):e0261487. doi: 10.1371/journal.pone.0261487 (PMC8675698; doi:10.1371/journal.pone.0261487)
Supplement: S1 Table — (DOC) [file pone.0261487.s005.doc]

S1 Table. Repetitive DNA sequence

| type | number | length | percent | |
| --- | --- | --- | --- | --- |
| ClassI/DIRS/DIRS | 2 | 125 | 0 | |
| ClassI/LINE/I | 50 | 142,526 | 0.43 | |
| ClassI/LTR/Copia | 584 | 1,683,450 | 5.07 | |
| ClassI/LTR/Gypsy | 994 | 3,889,792 | 11.72 | |
| ClassI/PLE/Penelope | 12 | 1,057 | 0 | |
| ClassII/Kolobok | 2 | 227 | 0 | |
| ClassII/Helitron | 33 | 89,706 | 0.27 | |
| ClassII/MITE | 65 | 25,156 | 0.08 | |
| ClassII/TIR | 267 | 484,046 | 1.46 | |
| ClassII/TIR/CACTA | 3 | 276 | 0 | |
| ClassII/TIR/PIF-Harbinger | 22 | 31,265 | 0.09 | |
| ClassII/TIR/Tc1-Mariner | 11 | 2,515 | 0.01 |  |
| ClassII/TIR/hAT | 2 | 275 | 0 | |
| Potential Host Gene | 18 | 80,825 | 0.24 | |
| Unknown | 779 | 671,665 | 2.02 | |
| Total with overlap: | 2,844 | 7,102,906 | 21.4 | |
| Total without overlap: | 2,844 | 7,033,311 | 21.19 | |
