# Supplementary material for: Genomic analysis of Elsinoë arachidis reveals its potential pathogenic mechanism and the biosynthesis pathway of elsinochrome toxin
Source: PLoS One. 2021 Dec 16;16(12):e0261487. doi: 10.1371/journal.pone.0261487 (PMC8675698; doi:10.1371/journal.pone.0261487)
Supplement: S4 Table — (DOCX) [file pone.0261487.s008.docx]

S4 Table. The loss of pathogenicity and reduced virulence genes in *E. arachidis*

| Loss of pathogenicity | PHI annotation | ID | Species |
| --- | --- | --- | --- |
| EVM0008698.1 | MgAlg2 | F9XJV5 | *Mycosphaerella graminicola* |
| EVM0001668.1 | CrzA | Q4WJ81 | *Aspergillus fumigatus* |
| EVM0005884.1 | MGG_00435 | EDK02952 | *Magnaporthe oryzae* |
| EVM0004309.1 | RHO1 | BAA24262 | *Candida albicans* |
| EVM0007481.1 | Mohik8 | G4MXJ1 | *Magnaporthe oryzae* |
| EVM0000123.1 | CLAP1 | AAN62846 | *Colletotrichum lindemuthianum* |
| EVM0000621.1 | BUF1 | MGG_02252 | *Magnaporthe oryzae* |
| EVM0004821.1 | cca1 | G4MNN4 | *Magnaporthe oryzae* |
| EVM0001657.1 | MoPEX7 | B8Q8Z5 | *Magnaporthe oryzae* |
| EVM0001881.1 | ABC3 | Q3Y5V5 | *Magnaporthe oryzae* |
| EVM0004199.1 | F-avi4330 | B9JV05 | *Agrobacterium vitis* |
| EVM0004124.1 | MoRic8 | XP_001405357 | *Magnaporthe oryzae* |
| EVM0004711.1 | BUF1 | MGG_02252 | *Magnaporthe oryzae* |
| EVM0007450.1 | AFT1 | BAB69076 | *Alternaria alternata* |
| EVM0003380.1 | AKT1 | BAA36588 | *Alternaria alternata* |
| EVM0001006.1 | AMT | AAF01762 | *Alternaria alternata* |
| EVM0001949.1 | VMA7 | EAL02110 | *Candida albicans* |
| EVM0001875.1 | MoVam7 | XP_001907727.1 | *Magnaporthe oryzae* |
| EVM0004857.1 | Calcium-transporting_ATPase_3 | MGG_02074 | *Magnaporthe oryzae* |
| EVM0008194.1 | MoSnf4 | G4NGW8 | *Magnaporthe oryzae* |
| EVM0007084.1 | PEX6 | AAK16738 | *Colletotrichum lagenarium* |
| EVM0007285.1 | Moatg16 | MGG_05255 | *Magnaporthe oryzae* |
| EVM0001939.1 | MoTea4 | G4N7A0 | *Magnaporthe oryzae* |
| EVM0000232.1 | CLA4 | AAT39367 | *Ustilago maydis* |
| EVM0003298.1 | MoSPC3 | G4MYT5 | *Magnaporthe oryzae* |
| EVM0007439.1 | ABC4 | MGG_00937 | *Magnaporthe oryzae* |
| EVM0006430.1 | CGB1 | AAO25585 | *Cochliobolus heterostrophus* |
| EVM0003095.1 | F-avi4330 | B9JV05 | *Agrobacterium vitis* |
| EVM0004079.1 | Con7p | Q069J4 | *Magnaporthe oryzae* |
| EVM0000759.1 | FAS2 | AAA34345 | *Candida albicans* |
| EVM0008892.1 | MgPex6 | G4NBI6 | *Magnaporthe oryzae* |
| EVM0002405.1 | Mohik8 | G4MXJ1 | *Magnaporthe oryzae* |
| EVM0003256.1 | UGD1 | AAK95561 | *Cryptococcus neoformans* |
| EVM0001667.1 | F-avi4330 | B9JV05 | *Agrobacterium vitis* |
| EVM0006405.1 | Mohik8 | G4MXJ1 | *Magnaporthe oryzae* |
| EVM0001244.1 | CAP59 | AAC13946 | *Cryptococcus neoformans* |
| EVM0005911.1 | snf7 | J9VU41 | *Cryptococcus neoformans* |
| EVM0004520.1 | ClaSSD1 | BAE66713 | *Colletotrichum lagenarium* |
| EVM0000474.1 | TOXF | AAD45321 | *Cochliobolus carbonum* |
| EVM0000135.1 | SOD5 | EAL00626 | *Candida albicans* |
| EVM0002950.1 | ABC4 | MGG_00937 | *Magnaporthe oryzae* |
| EVM0006879.1 | ClaSSD1 | BAE66713 | *Colletotrichum lagenarium* |
| EVM0004606.1 |  | MGG_04100 | *Magnaporthe oryzae* |
| EVM0004057.1 | Lmepi | B9DR51 | *Leptosphaeria maculans* |
| EVM0000747.1 | ABC3 | Q3Y5V5 | *Magnaporthe oryzae* |
| EVM0009019.1 | MET3 | AAL92174 | *Cryptococcus neoformans* |
| EVM0001193.1 | MGG_04587 | EDK05593 | *Magnaporthe oryzae* |
| EVM0003728.1 | CGB1 | AAO25585 | *Cochliobolus heterostrophus* |
| EVM0006401.1 | Moatg8 | MGG_01062 | *Magnaporthe oryzae* |
| EVM0004429.1 | Neo1 | MGG_04066 | *Magnaporthe oryzae* |
| EVM0007107.1 | TOXF | AAD45321 | *Cochliobolus carbonum* |
| EVM0001274.1 | F-avi4330 | B9JV05 | *Agrobacterium vitis* |
| EVM0002458.1 | AFT3 | BAB69078 | *Alternaria alternata* |
| EVM0003703.1 | ABC4 | MGG_00937 | *Magnaporthe oryzae* |
| EVM0001108.1 | AKT1 | BAA36588 | *Alternaria alternata* |
| EVM0002213.1 | ABC3 | Q3Y5V5 | *Magnaporthe oryzae* |
| EVM0006887.1 | MgAlg2 | F9XJV5 | *Mycosphaerella graminicola* |
| EVM0000043.1 | AFT1 | BAB69076 | *Alternaria alternata* |
| EVM0008972.1 | Moatg1 | MGG_06393 | *Magnaporthe oryzae* |
| EVM0008056.1 | PEX6 | AAK16738 | *Colletotrichum lagenarium* |
| EVM0005346.1 | VdSge1 | G2XD29 | *Verticillium dahliae* |
| EVM0000302.1 | URA5 | AAC62627 | *Histoplasma capsulatum* |
| EVM0002720.1 | Ubc2 | Q96X32 | *Ustilago maydis* |
| EVM0000780.1 | Gas1 | CAF05793 | *Ustilago maydis* |
| EVM0005164.1 | ABC3 | Q3Y5V5 | *Magnaporthe oryzae* |
| EVM0007245.1 | GLO1 | CAD79488 | *Ustilago maydis* |
| EVM0007013.1 | Neo1 | MGG_04066 | *Magnaporthe oryzae* |
| EVM0006906.1 | RUM1 | AAG02418 | *Ustilago maydis* |
| EVM0003814.1 | ILV2 | AAR29084 | *Cryptococcus neoformans* |
| EVM0000996.1 | BUF1 | MGG_02252 | *Magnaporthe oryzae* |
| EVM0003911.1 | PTH3 | AAB88888 | *Magnaporthe oryzae* |
| EVM0007487.1 | MGG_00435 | EDK02952 | *Magnaporthe oryzae* |
| EVM0003641.1 | RAS2 | AAO19639 | *Ustilago maydis* |
| EVM0007927.1 | VPS4 | Q5AG40 | *Candida albicans* |
| EVM0003974.1 | MoSnf4 | G4NGW8 | *Magnaporthe oryzae* |
| EVM0000488.1 | MoAtg4 | B6VCT6 | *Magnaporthe oryzae* |
| EVM0000582.1 | sskB | Q4WT11 | *Aspergillus fumigatus* |
| EVM0008978.1 | MGG_00883 | EDK02450 | *Magnaporthe oryzae* |
| EVM0005760.1 | ILV2 | AAR29084 | *Cryptococcus neoformans* |
| EVM0009121.1 | Gas1 | CAF05793 | *Ustilago maydis* |
| EVM0004701.1 | MGG_00435 | EDK02952 | *Magnaporthe oryzae* |
| EVM0005229.1 | CLAP1 | AAN62846 | *Colletotrichum lindemuthianum* |
| EVM0004737.1 | ABC3 | Q3Y5V5 | *Magnaporthe oryzae* |
| EVM0006532.1 | MGSTE11p | C6KEF4 | *Mycosphaerella graminicola* |
| EVM0008072.1 | F-avi4330 | B9JV05 | *Agrobacterium vitis* |
| EVM0006187.1 | MgRho3 | ABK60346 | *Magnaporthe oryzae* |
| EVM0002921.1 | SOD5 | EAL00626 | *Candida albicans* |
| EVM0006140.1 | AFT1 | BAB69076 | *Alternaria alternata* |
| EVM0005014.1 | Lmepi | B9DR51 | *Leptosphaeria maculans* |
| EVM0000903.1 | Moatg7 | MGG_07297 | *Magnaporthe oryzae* |
| EVM0007708.1 | TPS1 | MGG_03860 | *Magnaporthe oryzae* |
| EVM0005199.1 | PHI:2155\| | B8Q8Z5 | *Magnaporthe oryzae* |
| EVM0008152.1 | ABC4 | MGG_00937 | *Magnaporthe oryzae* |
| EVM0003047.1 | ABC3 | Q3Y5V5 | *Magnaporthe oryzae* |
| EVM0008999.1 | Moatg9 | MGG_09559 | *Magnaporthe oryzae* |
| EVM0001825.1 | MGG_00435 | EDK02952 | *Magnaporthe oryzae* |
| EVM0008019.1 | BUF1 | MGG_02252 | *Magnaporthe oryzae* |
| EVM0006918.1 | F-avi4330 | B9JV05 | *Agrobacterium vitis* |
| EVM0000860.1 | VdSge1 | G2XD29 | *Verticillium dahliae* |
| EVM0008540.1 | Moatg12 | MGG_00598 | *Magnaporthe oryzae* |
| EVM0006284.1 | RHO1 | BAA24262 | *Candida albicans* |
| EVM0000208.1 |  | MGG_04100 | *Magnaporthe oryzae* |
| EVM0009076.1 | Mls1 |  | *Phaeosphaeria nodorum* |
| EVM0004298.1 | CLNR1 | AAN65464 | *Colletotrichum lindemuthianum* |
| EVM0005519.1 | Neo1 | MGG_04066 | *Magnaporthe oryzae* |
| EVM0005638.1 | RHO1 | BAA24262 | *Candida albicans* |
| EVM0008620.1 | MGG_00435 | EDK02952 | *Magnaporthe oryzae* |
| EVM0003240.1 | Moatg7 | MGG_07297 | *Magnaporthe oryzae* |
| EVM0004942.1 | Lmepi | B9DR51 | *Leptosphaeria maculans* |
| EVM0008722.1 | F-avi4330 | B9JV05 | *Agrobacterium vitis* |
| EVM0000556.1 | ACL2 | I1RPR1 | *Gibberella zeae* |
| EVM0007753.1 | ACL1 | I1S7N4 | *Gibberella zeae* |
| EVM0003497.1 | snf7 | A0A095CFR8 | *Cryptococcus gattii* |
| EVM0006012.1 | MgAlg2 | F9XJV5 | *Mycosphaerella graminicola* |
| EVM0003124.1 | Mohik8 | G4MXJ1 | *Magnaporthe oryzae* |
| EVM0007228.1 | CGB1 | AAO25585 | *Cochliobolus heterostrophus* |
| EVM0004132.1 | F-avi4330 | B9JV05 | *Agrobacterium vitis* |
| EVM0008770.1 | Lmepi | B9DR51 | *Leptosphaeria maculans* |
| EVM0004954.1 | MgPex6 | G4NBI6 | *Magnaporthe oryzae* |
| EVM0001666.1 | ILV2 | AAR29084 | *Cryptococcus neoformans* |
| EVM0002336.1 | Moatg15 | MGG_12828 | *Magnaporthe oryzae* |
| EVM0007945.1 | CDC15 | XP_001406795 | *Magnaporthe oryzae* |
| EVM0001440.1 | Gas1 | CAF05793 | *Ustilago maydis* |
| EVM0002174.1 | SIDA | AAX40989 | *Aspergillus fumigatus* |
| EVM0005246.1 | ABC3 | Q3Y5V5 | *Magnaporthe oryzae* |
| EVM0005023.1 | RHO1 | BAA24262 | *Candida albicans* |
| EVM0000521.1 | BUF1 | MGG_02252 | *Magnaporthe oryzae* |
| EVM0006031.1 | CaCDC35 | AAG18428 | *Candida albicans* |
| EVM0005274.1 | ABC3 | Q3Y5V5 | *Magnaporthe oryzae* |
| EVM0006552.1 | CHK1 | O59892 | *Candida albicans* |
| EVM0008640.1 | TOXF | AAD45321 | *Cochliobolus carbonum* |
| EVM0005742.1 | LmIFRD | C6KED4 | *Leptosphaeria maculans* |
| EVM0005697.1 | Calnexin | MGG_01607 | *Magnaporthe oryzae* |
| EVM0001453.1 | galU | Q8P8Q1 | *Xanthomonas campestris* |
| EVM0004518.1 | PEX6 | AAK16738 | *Colletotrichum lagenarium* |
| EVM0002037.1 | CLAP1 | AAN62846 | *Colletotrichum lindemuthianum* |
| EVM0005077.1 | F-avi4330 | B9JV05 | *Agrobacterium vitis* |
| EVM0007501.1 | BMP1 | AAG23132 | *Botrytis cinerea* |
| EVM0004716.1 | CAP20 | AAA77678 | *Colletotrichum gloeosporioides* |
| EVM0003585.1 | Mohik5 | G4MKP6 | *Magnaporthe oryzae* |
| EVM0000271.1 | SOD2 | AAS19620 | *Cryptococcus gattii* |
| EVM0006795.1 | MGG_00435 | EDK02952 | *Magnaporthe oryzae* |
| EVM0005265.1 | SPT3 | AAD33888 | *Candida albicans* |
| EVM0000069.1 | MoSip2 | G4MNE3 | *Magnaporthe oryzae* |
| EVM0000042.1 | Mohik8 | G4MXJ1 | *Magnaporthe oryzae* |
| EVM0000685.1 | FSR1 | Q2TJF8 | *Gibberella moniliformis* |
| EVM0003970.1 | ZafA | ABJ98717 | *Aspergillus fumigatus* |
| EVM0006080.1 | Avenacinase_gene | AAB09777 | *Gaeumannomyces graminis* |
| EVM0008218.1 | MGG_00435 | EDK02952 | *Magnaporthe oryzae* |
| EVM0002331.1 | AFT1 | BAB69076 | *Alternaria alternata* |
| EVM0007987.1 | MgATG5 | EF486491 | *Magnaporthe oryzae* |
| EVM0004755.1 | CAP1 | AAD42978 | *Candida albicans* |
| EVM0003958.1 | ABC4 | MGG_00937 | *Magnaporthe oryzae* |
| EVM0002448.1 | TIG1 | MGG-03198.5 | *Magnaporthe oryzae* |
| EVM0008712.1 | Lmepi | B9DR51 | *Leptosphaeria maculans* |
| EVM0003917.1 | CDC15 | XP_001406795 | *Magnaporthe oryzae* |
| EVM0009117.1 | Calcium-transporting_ATPase_3 | MGG_05078 | *Magnaporthe oryzae* |
| EVM0002342.1 | Gas1 | CAF05793 | *Ustilago maydis* |
| EVM0003332.1 | AKT1 | BAA36588 | *Alternaria alternata* |
| EVM0006233.1 | UGD1 | AAK95561 | *Cryptococcus neoformans* |
| EVM0007069.1 | F-avi4330 | B9JV05 | *Agrobacterium vitis* |
| EVM0001190.1 | ABC3 | Q3Y5V5 | *Magnaporthe oryzae* |
| EVM0003192.1 | Gas1 | CAF05793 | *Ustilago maydis* |
| EVM0003985.1 | Gas1 | CAF05793 | *Ustilago maydis* |
| EVM0008531.1 | AFT1 | BAB69076 | *Alternaria alternata* |
| EVM0000782.1 | snf7 | A0A095CFR8 | *Cryptococcus gattii* |
| EVM0005623.1 | MGSTE7p | C5MK57 | *Mycosphaerella graminicola* |
| EVM0008013.1 | MoSip2 | G4MNE3 | *Magnaporthe oryzae* |
| EVM0001308.1 | UGD1 | AAK95561 | *Cryptococcus neoformans* |
| EVM0002415.1 | Annexin_A7 | MGG_06847 | *Magnaporthe oryzae* |
| EVM0001539.1 | Gas1 | CAF05793 | *Ustilago maydis* |
| EVM0003268.1 | AMT | AAF01762 | *Alternaria alternata* |
| EVM0006181.1 | HrpM | Q8PPR7 | *Xanthomonas citri ssp. Citri* |
| EVM0005231.1 | AFT1 | BAB69076 | *Alternaria alternata* |
| EVM0008962.1 | ABC3 | Q3Y5V5 | *Magnaporthe oryzae* |
| EVM0008739.1 | Moatg6 | MGG_03694 | *Magnaporthe oryzae* |
| EVM0006268.1 | PHI:451\| | AAC31119 | *Candida albicans* |
| EVM0001860.1 | CLAP1 | AAN62846 | *Colletotrichum lindemuthianum* |
| EVM0002548.1 | VPS4 | Q5AG40 | *Candida albicans* |
| EVM0005375.1 | MGG_00435 | EDK02952 | *Magnaporthe oryzae* |
| EVM0000738.1 | RPK1 | AAK31209 | *Colletotrichum lagenarium* |
| EVM0005630.1 | FOW2 | BAE98264 | *Fusarium oxysporum* |
| EVM0007959.1 | PTH2 | MGG_01099 | *Magnaporthe oryzae* |
| EVM0000516.1 | BUF1 | MGG_02252 | *Magnaporthe oryzae* |
| EVM0004495.1 | CDC24 | AAO25556 | *Candida albicans* |
| EVM0001578.1 | CMLE | H9C592 | *Fusarium oxysporum f. sp. Lycopersici* |
| EVM0008358.1 | VPS34 | CAA70254 | *Candida albicans* |
| EVM0002245.1 | F-avi4330 | B9JV05 | *Agrobacterium vitis* |
| EVM0008430.1 | AFT3 | BAB69078 | *Alternaria alternata* |
| EVM0008676.1 | MoSOM1 | XP_362263 | *Magnaporthe oryzae* |
| EVM0006149.1 | CMLE | H9C592 | *Fusarium oxysporum f. sp. Lycopersici* |
| EVM0000755.1 | FTR1 | AAF69680 | *Candida albicans* |
| EVM0005756.1 | CAP59 | AAC13946 | *Cryptococcus neoformans* |
| EVM0007973.1 | Mohik8 | G4MXJ1 | *Magnaporthe oryzae* |
| EVM0007088.1 | CPRGS-1 | AAT92283 | *Cryphonectria parasitica* |
| EVM0001820.1 | CaCDC35 | AAG18428 | *Candida albicans* |
| EVM0004258.1 | Avenacinase_gene | AAB09777 | *Gaeumannomyces graminis* |
| EVM0005690.1 | CPRGS-1 | AAT92283 | *Cryphonectria parasitica* |
| EVM0003421.1 | CGB1 | AAO25585 | *Cochliobolus heterostrophus* |
| EVM0005692.1 | MET3 | AAL92174 | *Cryptococcus neoformans* |
| EVM0000397.1 | ABC3 | Q3Y5V5 | *Magnaporthe oryzae* |
| EVM0005137.1 | Annexin_A7 | MGG_06847 | *Magnaporthe oryzae* |
| EVM0008732.1 | BUF1 | MGG_02252 | *Magnaporthe oryzae* |
| EVM0005228.1 | MGG_00435 | EDK02952 | *Magnaporthe oryzae* |
