# Supplementary material for: Genomic analysis of Elsinoë arachidis reveals its potential pathogenic mechanism and the biosynthesis pathway of elsinochrome toxin
Source: PLoS One. 2021 Dec 16;16(12):e0261487. doi: 10.1371/journal.pone.0261487 (PMC8675698; doi:10.1371/journal.pone.0261487)
Supplement: S5 Table — (DOCX) [file pone.0261487.s009.docx]

S5 Table. Increased virulence genes in *E. arachidis*

| Increased virulence | PHI annotation | ID | Species |
| --- | --- | --- | --- |
| EVM0007524.1 | Related_to_O-methylsterigmatocystin_oxidoreductase | I1R980 | *Gibberella zeae* |
| EVM0000012.1 | MfCUT1 | Q2VF46 | *Monilinia fructicola* |
| EVM0005728.1 | AKT7 | V5XZS6 | *Alternaria alternata* |
| EVM0000668.1 | Related_to_O-methylsterigmatocystin_oxidoreductase | I1R980 | *Gibberella zeae* |
| EVM0007404.1 | Rv2467 | L7N655 | *Mycobacterium tuberculosis* |
| EVM0001699.1 | AKT7 | V5XZS6 | *Alternaria alternata* |
| EVM0002472.1 | MeaB | J9MHC1 | *Fusarium oxysporum* |
| EVM0005577.1 | AKT7 | V5XZS6 | *Alternaria alternata* |
| EVM0005020.1 | Amr1 | G3F820 | *Alternaria brassicicola* |
| EVM0004619.1 | Related_to_O-methylsterigmatocystin_oxidoreductase | I1R980 | *Gibberella zeae* |
| EVM0000453.1 | AKT7 | V5XZS6 | *Alternaria alternata* |
| EVM0003003.1 | Related_to_O-methylsterigmatocystin_oxidoreductase | I1R980 | *Gibberella zeae* |
| EVM0007429.1 | ipa | B0LLU0 | *Leptosphaeria maculans* |
| EVM0001594.1 | ipa | B0LLU0 | *Leptosphaeria maculans* |
| EVM0006985.1 | Related_to_O-methylsterigmatocystin_oxidoreductase | I1R980 | *Gibberella zeae* |
| EVM0000714.1 | gacS | W0WL00 | *Pseudomonas aeruginosa* |
| EVM0005499.1 | AKT7 | V5XZS6 | *Alternaria alternata* |
| EVM0007024.1 | AKT7 | V5XZS6 | *Alternaria alternata* |
| EVM0000513.1 | Rv2467 | L7N655 | *Mycobacterium tuberculosis* |
| EVM0002803.1 | AKT7 | V5XZS6 | *Alternaria alternata* |
| EVM0000638.1 | Related_to_O-methylsterigmatocystin_oxidoreductase | I1R980 | *Gibberella zeae* |
| EVM0008001.1 | Rv2467 | L7N655 | *Mycobacterium tuberculosis* |
| EVM0008088.1 | Related_to_O-methylsterigmatocystin_oxidoreductase | I1R980 | *Gibberella zeae* |
| EVM0005295.1 | Related_to_O-methylsterigmatocystin_oxidoreductase | I1R980 | *Gibberella zeae* |
| EVM0001984.1 | AKT7 | V5XZS6 | *Alternaria alternata* |
| EVM0000505.1 | Related_to_O-methylsterigmatocystin_oxidoreductase | I1R980 | *Gibberella zeae* |
| EVM0007534.1 | AlHK1 | Q09JB7 | *Alternaria longipes* |
| EVM0005568.1 | Amr1 | G3F820 | *Alternaria brassicicola* |
| EVM0008524.1 | AlHK1 | Q09JB7 | *Alternaria longipes* |
| EVM0002495.1 | Related_to_O-methylsterigmatocystin_oxidoreductase | I1R980 | *Gibberella zeae* |
| EVM0007227.1 | AKT7 | V5XZS6 | *Alternaria alternata* |
| EVM0004574.1 | MfCUT1 | Q2VF46 | *Monilinia fructicola* |
| EVM0007009.1 | ampG | Q8P471 | *Xanthomonas campestris* |
| EVM0008731.1 | AKT7 | V5XZS6 | *Alternaria alternata* |
| EVM0006189.1 | AKT7 | V5XZS6 | *Alternaria alternata* |
| EVM0001742.1 | RsmA | D0KML5 | *Pectobacterium wasabiae* |
| EVM0007352.1 | Related_to_O-methylsterigmatocystin_oxidoreductase | I1R980 | *Gibberella zeae* |
| EVM0008331.1 | AKT7 | V5XZS6 | *Alternaria alternata* |
| EVM0006045.1 | ipa | B0LLU0 | *Leptosphaeria maculans* |
| EVM0007679.1 | AKT7 | V5XZS6 | *Alternaria alternata* |
| EVM0001119.1 | AKT7 | V5XZS6 | *Alternaria alternata* |
| EVM0005922.1 | AKT7 | V5XZS6 | *Alternaria alternata* |
| EVM0007377.1 | AKT7 | V5XZS6 | *Alternaria alternata* |
| EVM0005888.1 | PD0681 | Q87DK1 | *Xylella fastidiosa* |
| EVM0005669.1 | Related_to_O-methylsterigmatocystin_oxidoreductase | I1R980 | *Gibberella zeae* |
| EVM0000106.1 | SCD | F8R4X8 | *Metarhizium anisopliae* |
| EVM0007935.1 | Related_to_O-methylsterigmatocystin_oxidoreductase | I1R980 | *Gibberella zeae* |
| EVM0001783.1 | Related_to_O-methylsterigmatocystin_oxidoreductase | I1R980 | *Gibberella zeae* |
| EVM0000967.1 | AKT7 | V5XZS6 | *Alternaria alternata* |
| EVM0007154.1 | AKT7 | V5XZS6 | *Alternaria alternata* |
| EVM0004784.1 | AKT7 | V5XZS6 | *Alternaria alternata* |
| EVM0009154.1 | PKR1 | AAG30146 | *Cryptococcus neoformans* |
| EVM0000218.1 | AKT7 | V5XZS6 | *Alternaria alternata* |
| EVM0003036.1 | sidN | K7NCV2 | *Epichloe festucae* |
| EVM0000178.1 | PD0681 | Q87DK1 | *Xylella fastidiosa* |
| EVM0003547.1 | ROM2 | AAW45289 | *Cryptococcus neoformans* |
| EVM0007135.1 | AKT7 | V5XZS6 | *Alternaria alternata* |
| EVM0007435.1 | AKT7 | V5XZS6 | *Alternaria alternata* |
| EVM0008311.1 | MfCUT1 | Q2VF46 | *Monilinia fructicola* |
| EVM0001185.1 | AKT7 | V5XZS6 | *Alternaria alternata* |
| EVM0006074.1 | AKT7 | V5XZS6 | *Alternaria alternata* |
| EVM0006863.1 | PPOA | EAL89712 | *Aspergillus fumigatus* |
| EVM0001207.1 | AKT7 | V5XZS6 | *Alternaria alternata* |
| EVM0000559.1 | PspB_(not_PD0218) | Q87ET0 | *Xylella fastidiosa* |
| EVM0003200.1 | ipa | B0LLU0 | *Leptosphaeria maculans* |
| EVM0001988.1 | Ohmm | W5ZQ93 | *Beauveria bassiana* |
| EVM0000150.1 | AKT7 | V5XZS6 | *Alternaria alternata* |
| EVM0006471.1 | ipa | B0LLU0 | *Leptosphaeria maculans* |
| EVM0000654.1 | AKT7 | V5XZS6 | *Alternaria alternata* |
| EVM0003099.1 | AKT7 | V5XZS6 | *Alternaria alternata* |
| EVM0007063.1 | Can2 | AAZ30051 | *Cryptococcus neoformans* |
| EVM0003149.1 | HSP90 | AAA02743 | *Saccharomyces cerevisiae* |
| EVM0005125.1 | SCD | F8R4X8 | *Metarhizium anisopliae* |
| EVM0006983.1 | AKT7 | V5XZS6 | *Alternaria alternata* |
| EVM0007925.1 | RacA | A0JC80 | *Epichloe festucae* |
