# Supplementary material for: Genomic analysis of Elsinoë arachidis reveals its potential pathogenic mechanism and the biosynthesis pathway of elsinochrome toxin
Source: PLoS One. 2021 Dec 16;16(12):e0261487. doi: 10.1371/journal.pone.0261487 (PMC8675698; doi:10.1371/journal.pone.0261487)
Supplement: S7 Table — (DOCX) [file pone.0261487.s011.docx]

S7 Table. CAZymes in *E. arachidis* and compared genome

| **Fungal species** | **Glycoside Hydrolases (GHs)** | **Polysaccharide Lyases (PLs)** | **Carbohydrate Esterases (CEs)** | | **Glycosyl Transferases (GTs)** | | **Carbohydrate Binding Modules (CBMs)** | | **Auxiliary activities (AAs)** | **total** |
| --- | --- | --- | --- | --- | --- | --- | --- | --- | --- | --- |
| ***B. graminis*** | 61 | 0 | | 10 | | 56 | | 14 | 15 | 142 |
| ***N. crassa*** | 177 | 4 | | 22 | | 76 | | 42 | 43 | 322 |
| ***S. sclerotiorum*** | 220 | 5 | | 32 | | 89 | | 65 | 69 | 415 |
| ***B. cinerea*** | 229 | 9 | | 34 | | 95 | | 68 | 74 | 441 |
| ***F. graminearum*** | 251 | 21 | | 42 | | 103 | | 69 | 73 | 490 |
| ***M. oryzae*** | 268 | 5 | | 53 | | 105 | | 86 | 90 | 521 |
| ***E. arachidis*** | 271 | 16 | | 109 | | 114 | | 66 | 84 | 602 |
