# Supplementary material for: Genomic analysis of Elsinoë arachidis reveals its potential pathogenic mechanism and the biosynthesis pathway of elsinochrome toxin
Source: PLoS One. 2021 Dec 16;16(12):e0261487. doi: 10.1371/journal.pone.0261487 (PMC8675698; doi:10.1371/journal.pone.0261487)
Supplement: S8 Table — (DOC) [file pone.0261487.s012.doc]

S8 Table. PKS sequence information table

| toxin | Pathogen | GI | Gene ID |
| --- | --- | --- | --- |
| Elsinochrome | *Elsinoë australis* | PSK53765 | *Eapks* |
|  | *Shiraia* sp. | AIW00658 | *slf14* |
|  | *Elsinoë* sp. | PNS21559 | *CQ-2017a* |
| Aflatoxin | *Aspergillus parasiticus* | Q12053 | *PKSL* |
|  | *Aspergillus flavus* | AAS89999 | *PksA* |
| Cercosporin | *Cercospora nicotianae* | AAT69682 | *CnCTB1* |
|  | *Mycosphaerella coffeicola* | ADO14690 | *McCTB1* |
|  | *Cercospora beticola* | XP_023460065 | *CbCTB1* |
|  | Cercospora zeina | ARU80380 | *CzCTB1* |
| Melanin | *Colletotrichum lagenarium* | BAA18956 | *ClPKS1* |
|  | *Gibberella zeae* | AAU10633 | *GzPKS12* |
|  | *Aspergillus clavatus* | XP_001276035 | *AcPKSP* |
|  | *Xylaria* sp. | AAM93545 | *XyPKS* |
|  | *Nodulisporium* sp. | AAD38786 | *NoPKS* |
|  | *Sordaria macrospore* | CAM35471 | *SmPKS* |
| T-toxin | *C. heterostrophus* | AAB08104 | *ChPKS1* |
|  | *Bipolaris maydis* | N4WHA7 | *PKS2* |
|  | *Bipolaris maydis* | N4WHE3 | *PKS1* |
